# “I was hungry and you gave me food”: Religiosity and attitudes toward redistribution

**DOI:** 10.1371/journal.pone.0214054

**Published:** 2019-03-22

**Authors:** Gizem Arikan, Pazit Ben-Nun Bloom

**Affiliations:** 1 Department of Political Science, Trinity College Dublin, Dublin, Ireland; 2 Department of Political Science, The Hebrew University of Jerusalem, Jerusalem, Israel; Universidade Federal de Minas Gerais, BRAZIL

## Abstract

Current literature presents conflicting findings concerning the effect of religiosity on attitudes towards redistribution. This paper attempts to reconcile these findings by arguing that the belief and social behavior dimensions of religiosity affect support for redistribution via different mechanisms, and that these effects are moderated by state welfare generosity. Using multilevel path analysis models on data from the World Values Survey, we show that the effect of the religious belief on attitudes towards redistribution is mediated by competing personal orientations—prosocial values and conservative identification—while the religious social behavior dimension significantly decreases support for redistribution via increased levels of happiness. Lower levels of welfare generosity increase the positive effect of prosocial orientations and weaken the negative effect conservative identification, leading to positive or null indirect effect of religiosity. These findings show the importance of taking into account the multiple dimensions of religiosity and institutional context when studying the relationship between religion and redistribution attitudes.

## Introduction

The relationship between religion and redistribution is admitted to be highly complex [1, 2], presenting conflicting theories and inconsistent empirical findings. Some researchers credit religiosity with justifying economic inequalities and defending the pro-market status quo [3–9]. At the same time, others suggest that religious beliefs are associated with concern for social and economic justice, as all the world’s major religious traditions espouse notions of fairness and helping others [10–12]. Yet, little research has attempted to reconcile these conflicting views or determine whether and to what extent religious prosociality translates into support for redistribution [7] (but see [13]).

**Fig 1:**
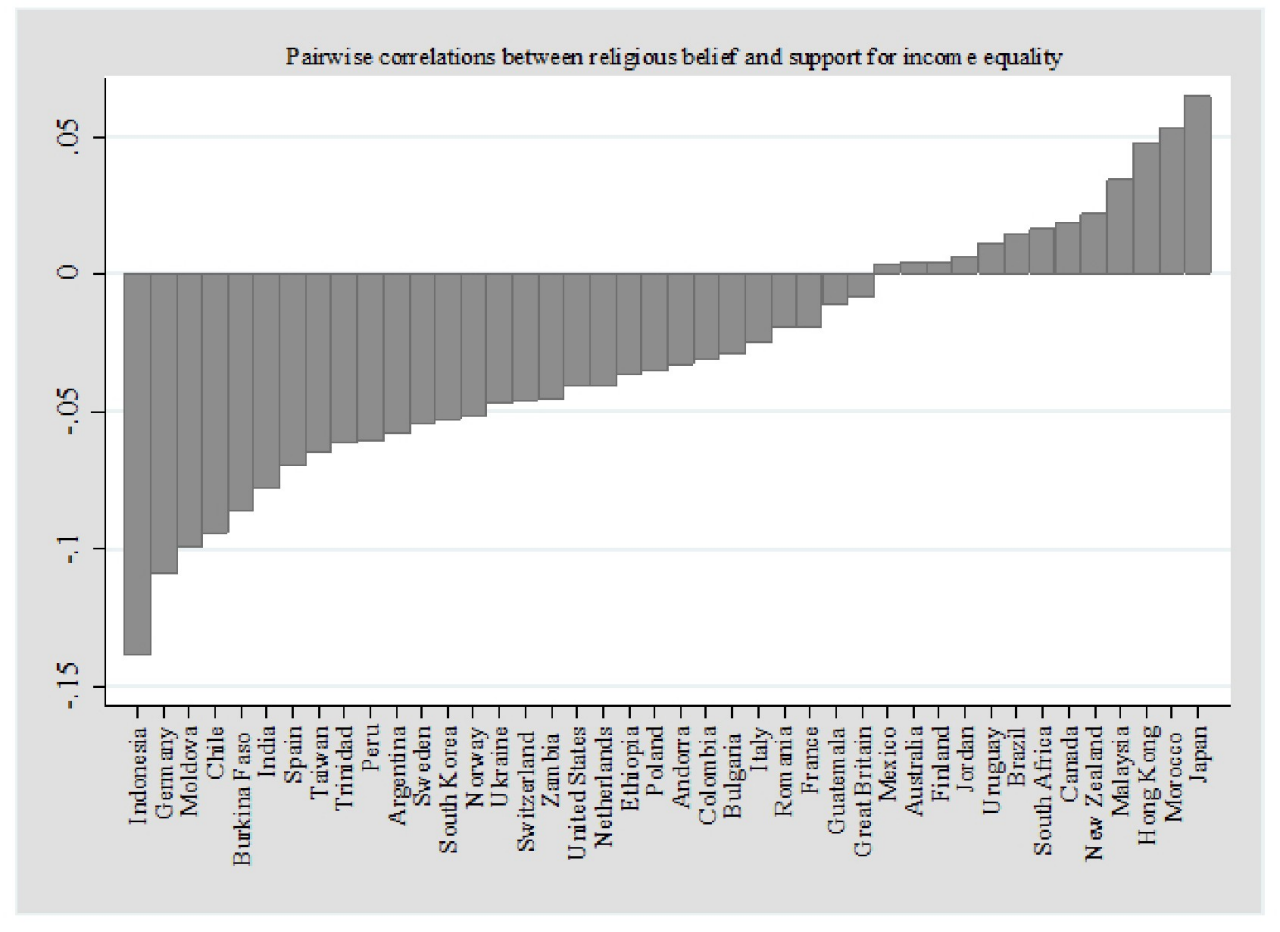
Pairwise correlations between religious belief and support for income inequality in selected countries included in the dataset. Entries are pairwise correlations calculated using data from World Values Survey (WVS) Wave 5.

Furthermore, research examining the effect of religiosity on redistribution attitudes has often presented contradictory findings, possibly because different studies capture different aspects of individual religious experience by using different indicators of religiosity. Although religiosity is increasingly theorized as a complex, multidimensional phenomenon [14, 15], existing empirical work has paid insufficient attention to the complex nature of individual religious orientations (but see [8, 13]). Secondly, most studies only focus on one or a few countries or religions [7, 8, 16–20]. It is therefore possible that some of the contradictory findings in the literature may be due to contextual influences. Fig 1, which presents individual-level pairwise correlations between religious beliefs and support for income equality in some countries included in the World Values Survey (WVS) Wave 5, shows that countries vary widely in the relationship between religious belief and support for the statement, “Incomes should be made more equal,” from substantively negative (r = -.13) to substantively positive (r = .06) values. This variability may be related to differences in religious orientations of respondents as well as contextual factors.

Currently, the literature on religion and redistribution lacks a theory that takes into account both the complexity of individual religiosity and the influence of contextual factors. This paper rectifies this by simultaneously considering multiple dimensions of individual-level religiosity, the psychological mechanisms that underlie them as well as country-level contextual factors. We suggest that two dimensions of religiosity—religious belief and religious social behavior—may have differential effects on support for redistribution. First, we claim that both religiously promoted prosocial values and conservative political identification mediate the effect of religious belief on attitudes toward redistribution, but in opposite directions. That is, while prosocial values increase support for redistribution, conservative identification decreases it. We claim that the social dimension of religiosity, religious social behavior, decreases support for redistribution by providing a psychological safety net that increases individual happiness. Second, we hypothesize that state welfare generosity influences the effect of the mediating mechanisms. In countries with higher levels of welfare benefits, the positive mediating effect of prosocial values will be reduced whereas the negative mediating effect of conservative identification will be higher, making the overall effect of religious belief on support for redistribution negative. Conversely, in countries where the welfare state is less generous, the positive mediating effect of prosocial orientations may override the negative mediating effect of conservative identification, thus leading to an overall positive effect of religious belief on attitudes towards redistribution.

We test our hypotheses using multilevel path analysis models on data from 49 democratic countries from Wave 5 of the World Values Surveys. Overall, our findings help explain the current mixed results in the field, showing that the generosity of state welfare influences the way religious beliefs affect attitudes towards redistribution. The results also suggest that considering only a single dimension of religiosity or focusing on a single context may prevent researchers from understanding the full complexity of the relationship between religion and attitudes towards redistribution.

## Religious belief: The competing effects of prosocial orientations and conservative identification

Individual religiosity is often conceptualized as a complex phenomenon consisting of belief, behavior, and belonging dimensions [14]. While these dimensions are closely related, recent research suggests that they may have different and even conflicting effects on political attitudes and behavior because each dimension underlies the effects of different psychological mechanisms [21]. For example, while religious belief does not have any significant impact on volunteerism or charitable activities, religious social behavior increases individual tendencies to participate in both types of activities [22]. Similarly, the belief dimension of religiosity has been shown to be associated with greater levels of support for immigration whereas the social behavior dimension generally has the opposite effect [23]. Differential effects of belief and social behavior have also been found with regards to voting behavior [24], protest participation [25], and attitudes towards climate change [26]. Because different psychological mechanisms underlie the effect of each dimension of religiosity, aggregating all dimensions of religious orientation into one broad concept may be misleading both theoretically and methodologically. Debates concerning the relationship between religiosity and attitudes towards redistribution also suggest that the effect of each dimension should be considered separately.

We start with the religious belief dimension, which relates to the content of an individual’s faith or belief in God, heaven, hell, and life after death [14, 15, 27]. The first way that intensity of religious beliefs may increase support for redistribution through promoting prosocial values. Religion is associated with the well-being of the community and with altruism and giving. All major religious faiths prescribe some degree of charity as in the parables of Christ, the Jewish tradition of *tzedaka*, and the Islamic pillar of *Zakat*” [11] and promote helping others by fostering prosocial values like benevolence and compassion toward the needy [28–31]. Churches and religiously established charities have historically been associated with helping the poor, the elderly, and the downtrodden [32–34] and religious people are found to donate more to charities [12, 35]. Indeed, there is both experimental and survey evidence for the positive effect of religious beliefs on prosociality across cultures and religious traditions [10, 30, 36]. These religiously inspired prosocial values can be expected to increase support for policies that aim at redistribution of income in society [13, 37].

**Fig 2:**
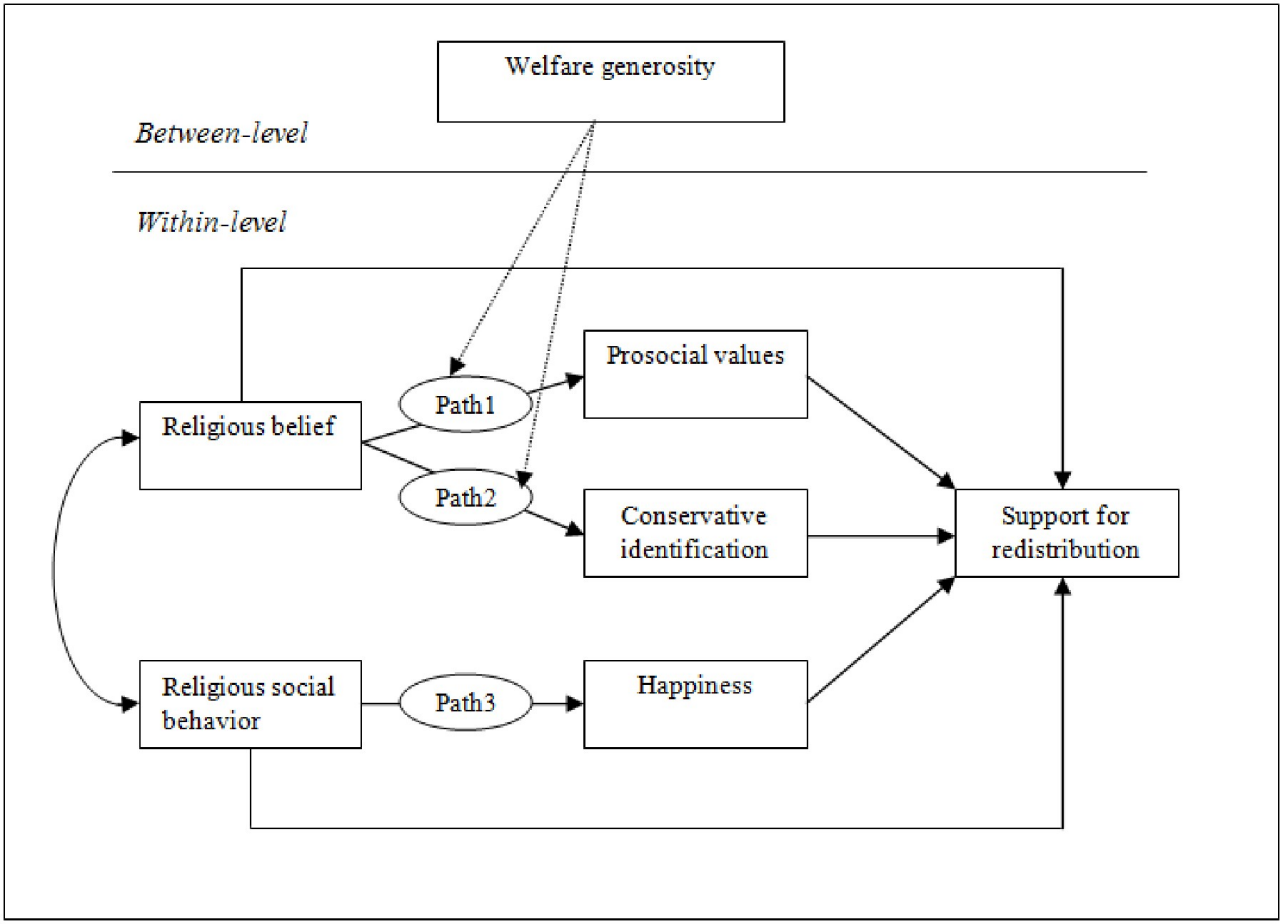
The proposed model.

A second potential mediator between religious belief and support for redistribution is conservative identification. Religious beliefs increase identification with conservative worldviews as well as support for conservative parties and ideologies [18, 38]. Religious beliefs may promote conservative identification partly because of shared assumptions concerning the causes of poverty and inequality, and an antipathy to big government. Regarding poverty and inequality, both Protestant Christianity and Islam emphasize individualism and value achievement, hard work, and self-reliance [3, 39]. This encourages believers from both religious traditions to accept structural inequalities, based on the view that poverty results from individual failure. Regarding governments, believers may see big governments as a threat to the power and prestige of religious institutions [6, 8, 9, 17, 34, 40]. In turn, those who champion individualism and perceive big government as a threat to religion tend to identify more with conservative political platforms [34, 38, 41]. Religious beliefs may also increase the tendency to identify with conservative worldviews because believers find common cause with social conservatism [13, 42]. Since most conservative ideologies bundle together positions that are socially and economically conservative, such as promoting self-help and pro-market policies [13, 18, 40, 42], they tend to discourage their adherents from supporting redistributive policies [13]. This observation also suggests that religious beliefs are positively associated with conservative political orientations. We therefore predict that conservative identification is associated with stronger opposition to redistributive policies. This leads us to hypothesize two mediating effects that pull the effects of religious belief on support for redistribution policies in opposite directions:

*Prosocial values positively mediate the effect of religious belief on support for redistribution (H1) whereas conservative identification negatively mediates the effect of religious belief on support for redistribution (H2)*. These two mediating effects are represented by paths 1 and 2 in Fig 2, which summarizes the proposed model and the hypotheses.

## Religious social behavior: Religious involvement as psychological insurance

Religious social behavior refers to participation in social religious activities and involvement in religious networks. The political economy literature suggests that the psychological security provided by participating in religious social activities reduces demand for government redistribution as a collective insurance device [4, 5, 8, 9, 43]. We suggest that the happiness resulting from communal religious activities mediates the effect of religious social behavior on attitudes towards redistribution. Research indicates that religious social behavior may provide both material and psychological benefits. Even if religious organizations may provide both material and psychological security, we expect these effects to work through the same psychological mechanism—that is, one’s happiness. In fact, studies show that the psychological insurance provided by religious organizations might be more relevant. For example, Clark and Lelkes [4] find that church participation has stress-buffering effects on individuals experiencing unemployment or marital separation while DeLeire and Kalil [44] show that material consumption does not necessarily have significant positive effects on happiness. Similarly, Chaves [45] notes that benefits provided by religious organizations generally emphasize cultural more than material objectives.

According to terror management theory [46], people cope with the awareness of their mortality by investing in cultures, such as religious communities, that offer symbolic immortality [47, 48]. That is, religious social behavior offers psychological security and well-being by affirming one’s membership of a group that outlives its members, thereby reducing anxiety and increasing happiness [47, 48]. Religious social behavior has a robust effect on positive emotions like happiness [4, 49–51] and reduces the negative effects of income shocks or unemployment on the happiness and life satisfaction of members of religious organizations [4, 5]. For religious individuals, their greater levels of happiness due to the psychological insurance afforded by their participation in a religious community makes them assess economic conditions more favorably [43]. In fact, well-being and happiness is associated with more favorable impressions and judgements [52–56] including political and economic evaluations. Happier individuals report greater confidence in government institutions [57, 58] and tend to evaluate the state of the economy more positively [59]. Perceptions of well-being in turn influence individuals’ responses to economic incentives and their attitudes towards the market economy with happier individuals being more likely to support market economy [59]. Individuals who are satisfied with their economic condition are generally less supportive of government redistribution [60] while those who feel that they are economically vulnerable are more supportive [61]. Happiness is also positively associated with perceived social mobility which reduces support for redistribution and increases support for market economy [62, 63]. This is because those who overestimate the level of social mobility in their society assume that they may become the rich of tomorrow and they do not want to be the ones who will have to support redistributive schemes [62]. Thus, we expect higher levels of happiness to erode support for redistributive policies [16, 60]. This leads us to propose the following hypothesis (shown as path 3 in Fig 2):

*Happiness negatively mediates the effect of religious social behavior on support for redistribution (H3)*.

The third dimension of religious experience, religious belonging refers to identification as a member of a particular organized denomination, movement, or trend within a denomination [64, 65]. Our study aims at examining whether the hypothesized psychological mechanisms extends beyond a single religious tradition or context. We expect the mechanisms to hold across members of different religious traditions given that the major traditions all provide a strong source of group identification [30] and emphasize compassion and caring for those who are in need [30, 36] and given the almost-universal relationship between religious beliefs and conservative political identifications [38], and between religious social behavior and happiness [66]. Still, in the robust analysis section we discuss a number of tests we conducted to evaluate to what extent the proposed psychological mechanisms held for members of different religious traditions.

## Contextual influences: The moderating effect of state welfare generosity

Scholars have long acknowledged that country-level institutional context, particularly redistribution arrangements, influence individual-level redistribution attitudes [67]. In the same vein, we argue that the mediating effects of prosocial and conservative orientations are conditional on the generosity of each country’s welfare state. The two arrows from welfare generosity to paths 1 and 2 in Fig 2 represent the following hypotheses.

First, we predict that higher levels of state welfare generosity weaken the positive effect of religious belief on prosocial orientations. This expectation is based on evidence that religion and social spending are alternative mechanisms of insurance [43]. State welfare provision decreases demand for the social security provided by religious institutions and communities [4, 43, 68]. Conversely, lower levels of state welfare provision increase demand for religious organizations to provide social services [17, 69]. Even in countries where religion and state are officially separated, a lack or insufficiency of government welfare provision stimulates demand for welfare provision from religious organizations [5, 17, 69].

We suggest that this mechanism influences the relationship between religious belief and prosocial orientations. When government provision of welfare proves inadequate, religious groups or organizations take on a more active role, either by directly providing welfare services themselves or by calling on the government to provide more [17, 70, 71]. Such religiously-motivated welfare provision helps to reinforce the link between religious beliefs and prosocial orientations because the providers base their discourse and mobilization strategies on appeals to religious values of compassion and a moral obligation to help others [17, 72]. In this way, the relationship of religious beliefs to prosocial values becomes much more emphasized. In contrast, social welfare systems are founded on the notions of rights, emancipation, and entitlement [72], which downplay the religious and moral aspects of providing welfare [8]. Thus, as state welfare generosity increases, we predict that the relationship between religion and prosocial orientations will become less explicit, and that the positive effect of religious belief on prosocial values will be reduced. This leads to the following hypothesis:

*The positive effect of religious belief on prosocial values decreases as state welfare generosity increases (H4)*.

Second, we predict that the relationship between religious belief and conservative identification becomes stronger as state welfare generosity increases. Research shows that, when the implications of policies are clear, citizens can more easily translate their pre-existing orientations into policy preferences [73]. Conversely, a lack or unreliability of information may make it hard for individuals to connect their values to policy preferences. According to Bartels [74], this is why most Americans tended to support the 2001 and 2003 tax cuts that ended up benefiting wealthy taxpayers. According to Gingrich [75], higher levels of welfare provision lead to greater levels of policy salience and create an informational environment that makes individuals more easily connect their basic preferences to policies and party choices [75]. Generous welfare policies may help individuals connect values of individualism and hard work to conservative ideologies. State welfare generosity may also make the religion-state conflict clearer to religious believers, helping them more easily link religiosity with conservative political orientations. This leads to our next hypothesis:

*The effect of religious belief on conservative identification increases as state welfare generosity increases (H5)*.

## Data and variables

We used data from 49 democratic countries in Wave 5 of the WVS. We excluded from the analysis countries classified by Freedom House as non-democracies (i.e. the “not free” category reserved for countries with scores between 5.5 and 7 in the Freedom Ratings). This excludes China, Iran, Iraq, Russia, Rwanda, Vietnam, Thailand, and Egypt from the analyses. This is since individuals with no experience of democratic forms of governance might have qualitatively different evaluations of institutions such as the welfare system, as they may not have access to reliable assessments and evaluations of public policies, spending, and political performance [21]. Since the measures for the key moderator variable, state welfare generosity, and for some control variables, such as religious fractionalization at the country-level, are missing for some countries, models that include these variables have fewer cases due to listwise deletion.

Support for redistribution was measured with the survey item that taps attitudes towards income equality in society and was treated as a continuous variable, in line with the previous studies that used this WVS item to measure redistribution attitudes [6, 60, 76–80]. Respondents were asked to place their views on a 1–10 scale where 1 indicates complete agreement with the statement: “Incomes should be made more equal,” and 10 indicates complete agreement with the statement: “We need larger income differences as incentives for individual effort.” We recoded the responses so that higher values represent greater support for income equality.

Regarding the independent variables, religious belief was an additive index of two items: whether the respondent considered herself a religious person (recoded so that 1 indicated religious and 0 otherwise) and to what extent the respondent characterized the importance of God in her life. This item was originally measured on a 10-point scale that ranged between 1 (not at all important) and 10 (very important). We rescaled this item to vary between 0 and 1. The additive religious belief item was then created by adding the two items, giving equal weight to each recoded item. Previous work compared this additive measure to a more complex measure of religious belief constructed from a larger number of religious belief items that were available in the earlier waves of WVS, and found that the two measures were highly correlated (r = 0.89). This provides support for the validity of the additive index, which we also used in our analyses [21].

Religious social behavior was an additive index of two items: The first item was the frequency of attending religious services, measured with a 7-point scale and rescaled to vary between 0 and 1 where higher values show more frequent attendance. The second item was whether the respondent was a member of a religious organization. We recoded the original responses such that 1 indicated no membership in such organizations and 0 otherwise. In line with previous studies [21, 81], the index was constructed by giving equal weight to both items.

Prosocial values were measured using Schwartz’s self-transcendence values coded from the shorter version of the Portrait Values Questionnaire available in the WVS. Social psychologist Shalom Schwartz has identified ten universal individual-level value orientations that form two higher-order dimensions representing two fundamental conflicts in human societies: openness to change versus conservation and self-transcendence versus self-enhancement [82]. The first higher-order value dimension contrasts the motivational goals of tradition, conformity and security with values representing a preference for individual autonomy: self-direction, stimulation and hedonism. The second higher order dimension contrasts self-enhancement values that emphasize the pursuit of self-interest by seeking to control people and resources (power) or by seeking success (achievement) and hedonism with self-transcendence values that emphasize concern for the well-being of others (benevolence) and acceptance and tolerance (universalism). In line with the instructions provided by Schwartz [83, 84], we first centered all ten values to control for “individual as well as group biases in use of the response scales” [83]. To do this, we first computed each individual’s mean score over all the value items. We then computed the centered scores of the values by subtracting this score from the individual scores [83, 84]. Next, and again in line with the standard procedure for coding Schwartz’s values, we added the centered values for universalism and benevolence to construct a measure of self-transcendence, as these are the two items that make up the higher-order self-transcendence dimension. We also created the values for the higher-order self-enhancement dimension by adding the three value items that make up this dimension: power, achievement, and hedonism. Since researchers are often interested in the relative priority attached to one value dimension over the other [84–86], (in this case, self-transcendence over self-enhancement), we subtract each respondent’s score on self-direction values from self-transcendence values. This final value is our measure for prosocial orientations.

Conservative political orientations were measured with the 1–10 left-right identification question where 10 represents the extreme right and 1 represents the extreme left [13]. Happiness was measured using a 4-category item that asked respondents to what extent they feel happy ranging from “very happy” to “not at all happy.” [5]. We recoded the variable such that higher values represent higher levels of happiness.

All the resulting measures and indices were rescaled to vary between 0 and 1. This rescaling facilitates the interpretation of findings such that all coefficients presented in the tables can be interpreted as the change in the value of dependent variable when moving from the minimum to the maximum value of the independent variable.

The moderator variable, welfare generosity, was measured with the Social Security Laws Index (SSLI) from the Regulation of Labor dataset [87]. SSLI measures the generosity of each country’s old age, disability, sickness, unemployment, and death benefits. One of the main advantages of this measure is that it is based on the laws and regulations that protect individuals against various life risks rather than spending measures, which generally depend on each country’s level of economic development [8, 75]. In addition, data is available for a wide range of countries, which increases the degrees of freedom and provides a wider range for key level-2 variables compared to welfare spending measures that are only available for a restricted number of countries.

Finally, the robust analyses specify individual level and country-level controls. Individual level controls include age (in years), gender (coded 1 if respondent was male, 0 otherwise), and level of education (coded 1 if the respondent had low education, and 0 otherwise). In addition, we control for GDP per capita (measured in PPP, logged) as an indicator of level of socioeconomic development, as well as religious fractionalization at the country-level.

Data sources, question wordings for survey items, and descriptive statistics are presented in S1 Table.

## Methods

To test our hypotheses, we used path analysis, a variant of Structural Equation Modeling (SEM) [88]. Path analysis is a causal modeling method that represents hypotheses about effect priority [89], making it suitable for testing whether the effects of religious belief and religious social behavior on support for redistribution are explained by the study’s mediator variables: prosocial values, conservative identification, and happiness.

Since our data consists of individuals nested within countries, we used a multilevel path model in which the within-cluster and between-cluster effects are estimated separately in a single analysis [90]. We model religious belief and religious social behavior as exogenous individual-level variables that are correlated with each other (see the arrow on the left of Fig 2 connecting the two variables). It is standard practice in SEM to include correlations between the independent or exogenous variables [88, 89]. In addition, in our case, we have theoretical reasons for assuming that religious belief and religious social behavior are correlated with each other. These correlations between the independent variables are estimated by the computer software, but they are unanalysed in the sense that no prediction is put forward about why these two variables covary such as whether one causes the other or whether they have a common cause [89].

We also specify direct effects of belief and social behavior dimensions on support for redistribution. These are represented by the arrows going directly from religious belief and religious social behavior to support for redistribution in Fig 2. Direct effects show the effects of the exogenous variables on the dependent variable once the effects of mediators are taken into account. As per H1, H2, and H3, the effects of religious belief and religious social behavior are partly explained by the mediator variables: prosocial values, conservative identification, and happiness. As a result, religious belief predicts prosocial values (Path 1 in Fig 2) and conservative identification (Path 2 in Fig 2) which also predict attitudes towards redistribution, and religious social behavior predicts happiness (Path 3 in Fig 2), which is also a predictor of redistribution attitudes. The indirect effect of each mediator would be calculated as a multiplication function of the effect of the exogenous variable on that mediator (thus, for example, the coefficient for Path 1 for prosocial values) and the effect of the mediator on the dependent variable (represented by the arrow linking prosocial values to the dependent variable). The two arrows linking welfare generosity to Path 1 and Path 2 in Fig 2 show the conditional effect of the level-2 variable, Social Security Laws Index on these paths as per H4 and H5 respectively. S1 File presents all the equations underlying models presented in Tables 1 and 2.

**Table 1:** Support for redistribution: Multilevel path models.

	Model 1.1	Model 1.2	Model 1.3	Model 1.4
**Within-level part of model**				
***Rel*. *belief mediators***				
Religious belief → Prosocial values	**.027 (.014)**	.*028 (*.*015)*	**.029 (.014)**	**.030 (.015)**
Religious belief → Conservative identification	**.133 (.022)**	**.144 (.023)**	**.136 (.021)**	**.148 (.021)**
***Religious social behavior mediators***				
Religious social behavior → Happiness	**.059 (.018)**	**.060 (.019)**	**.064 (.017)**	**.064 (.019)**
Religious belief → DV	*-*.*143 (*.*083)*	*-*.*156 (*.*093)*	**-.223 (.082)**	**-.234 (.091)**
Prosocial values → DV	**.708 (.293)**	**.676 (.329)**	**.611 (.291)**	**.631 (.326)**
Conservative identification → DV	**-1.254 (.175)**	**-1.266 (.187)**	**-1.293 (.167)**	**-1.306 (.179)**
Religious social behavior → DV	-.057 (.067)	-.046 (.072)	-.065 (.064)	-.051 (.069)
Happiness → DV	**-.643 (.146)**	**-.629 (.148)**	**-.548 (.117)**	**-.535 (.138)**
Corr. (Religious belief, religious social behavior	**.058 (.007)**	**.057 (.008)**	**.058 (.007)**	**.057 (.008)**
***Individual-level controls***				
Age → Prosocial values	-	-	**.002 (.000)**	**.002 (.000)**
Gender (Male = 1) → Prosocial values	-	-	**-.024 (.003)**	**-.025 (.003)**
Low education (dummy) → Prosocial values	-	-	**-.012 (.005)**	*-*.*011 (*.*006)*
Age → Conservative identification	-	-	.000 (.000)	.000 (.000)
Gender (Male = 1) → Conservative identification	-	-	**.017 (.004)**	**.019 (.004)**
Low education (dummy) → Conservative identification	-	-	-.001 (.011)	-.003 (.012)
Age → Happiness	-	-	**-.001 (.000)**	**-.001 (.000)**
Gender (Male = 1) → Happiness	-	-	-.001 (.004)	.001 (.004)
Low education (dummy) → Happiness	-	-	**-.048 (.011)**	**-.050 (.011)**
Age → DV	-	-	-.001 (.002)	-.002 (.002)
Gender (Male = 1) → DV	-	-	**-.139 (.029)**	**-.145 (.033)**
Low education (dummy) → DV	-	-	**.553 (.046)**	**.563 (.050)**
**Between-level part of model**				
Social Security Laws Index	-	*1*.*746 (*.*901)*	-	**1.773 (.835)**
GDP per capita (PPP), logged	-	.158 (.195)	-	.194 (.183)
Religious fractionalization	-	-.352 (.449)	-	-.246 (.439)
**Model fit statistics**				
CFI / TLI / RMSEA	.982/.954/.006	.983/.948/.007	.933/.816/.011	.946/.837/.010
Chi2 model fit for baseline model (d.f.) / p-value	889.241 (15) / .0000	895.297 (18) / .0000	1346.825 (33) / .0000	1205.400 (36) / .0000
N1/N2	65980 / 49	55681 / 40	65278 / 49	55028 / 40

Entries are coefficients with robust standard errors in brackets. Italic entries indicate p < 0.1 (two-tailed) and bold entries indicate p < 0.05 (two-tailed).

**Table 2:** Moderation of paths 1 and 2: M-SEM analysis.

	Model 2.1	Model 2.2	Model 2.3	Model 2.4
**Within-level part of model**				
***Rel*. *belief mediators***				
Prosocial values → DV	**.632 (.306)**	**.685 (.312)**	.*576 (*.*305)*	**.630 (.311)**
Conservative identification → DV	**-1.280 (.187)**	**-1.311 (.195)**	**-1.319 (.179)**	**-1.347 (.187)**
Religious belief → DV	**-.214 (.083)**	**-.216 (.087)**	**-.298 (.084)**	**-.305 (.087)**
***Individual-level controls***				
Age → Prosocial values	**-**	**-**	**.002 (.000)**	**.002 (.000)**
Gender (Male = 1) → Prosocial values	**-**	**-**	**-.022 (.003)**	**-.022 (.003)**
Low education (dummy) → Prosocial values	**-**	**-**	**-.008 (.002)**	**-.008 (.002)**
Age → Conservative identification	**-**	**-**	**.001 (.000)**	**.000 (.000)**
Gender (Male = 1) → Conservative identification	**-**	**-**	**.013 (.004)**	**.015 (.004)**
Low education (dummy) → Conservative identification	**-**	**-**	**.019 (.008)**	**.018 (.009)**
Age → DV	**-**	**-**	-.001 (.002)	-.001 (.002)
Gender (Male = 1) → DV	**-**	**-**	**-.142 (.033)**	**-.147 (.034)**
Low education (dummy) → DV	**-**	**-**	**.582 (.050)**	**.594 (.051)**
**Between-level part of model**				
SSLI → Path 1	**-.094 (.033)**	**-.092 (.033)**	**-.118 (.035)**	**-.116 (.035)**
SSLI → Path 2	.056 (.066)	.072 (.065)	.044 (.065)	.062 (.064)
SSLI → Prosocial values	**.195 (.031)**	**.195 (.031)**	**.161 (.033)**	**.162 (.032)**
SSLI → Conservative identification	-.043 (.072)	-.076 (.068)	-.037 (.074)	-.068 (.070)
SSLI → DV	**2.476 (.484)**	**1.846 (.921)**	**2.607 (.494)**	**1.792 (.854)**
GDP per capita (PPP), logged → DV	-	.184 (.208)	-	.232 (.198)
Religious fractionalization → DV	-	-.216 (.387)	-	-.302 (.363)
**Model Fit Indices**				
-2 x Log likelihood	199853.54	191835.74	194300.59	186527.27
AIC	199901.54	191887.74	194366.59	186597.27
BIC	200115.36	192118.28	194660.26	186907.25
n-adj. BIC	200039.09	192035.65	194555.39	186796.02
N (Level 1 / Level 2)	54680/ 42	54413 / 40	54126 / 42	51878 / 40

Entries are coefficients with robust standard errors in brackets. Italic entries indicate p < 0.1 (two-tailed) and bold entries indicate p < 0.05 (two-tailed).

Path models intend to represent the most parsimonious explanation for the model under question. Therefore path models ideally use relatively few variables and paths [91] (for applications see for example [13, 21, 92]. Nevertheless, some of the models below specify additional controls to examine whether the results are robust to the inclusion of demographic variables. We specify age, gender, and level of education as additional exogenous variables predicting the three mediators as well as the dependent variable (See [93] for this strategy, and [94] for a similar application). We run the analysis first of the most parsimonious model (Model 1.1; as indicated in Fig 2) and then start adding the individual and country level controls (Models 1.2, 1.3, and 1.4).

MPLUS 6.1 is used to run the analysis. The software calculates the standard errors and chi-square test of model fit correcting for the non-independence of observations due to clustering and does not rely on the homoscedasticity assumption [95, 96]. Therefore results presented in Tables 1 and 2 below present robust standard errors. We evaluate the results by focusing on model fit, path coefficients, and their standard errors (shown in Tables 1 and 2), as well as direct and indirect effects and their standard errors (shown in S2 and S3 Tables).

## Results

**Fig 3:**
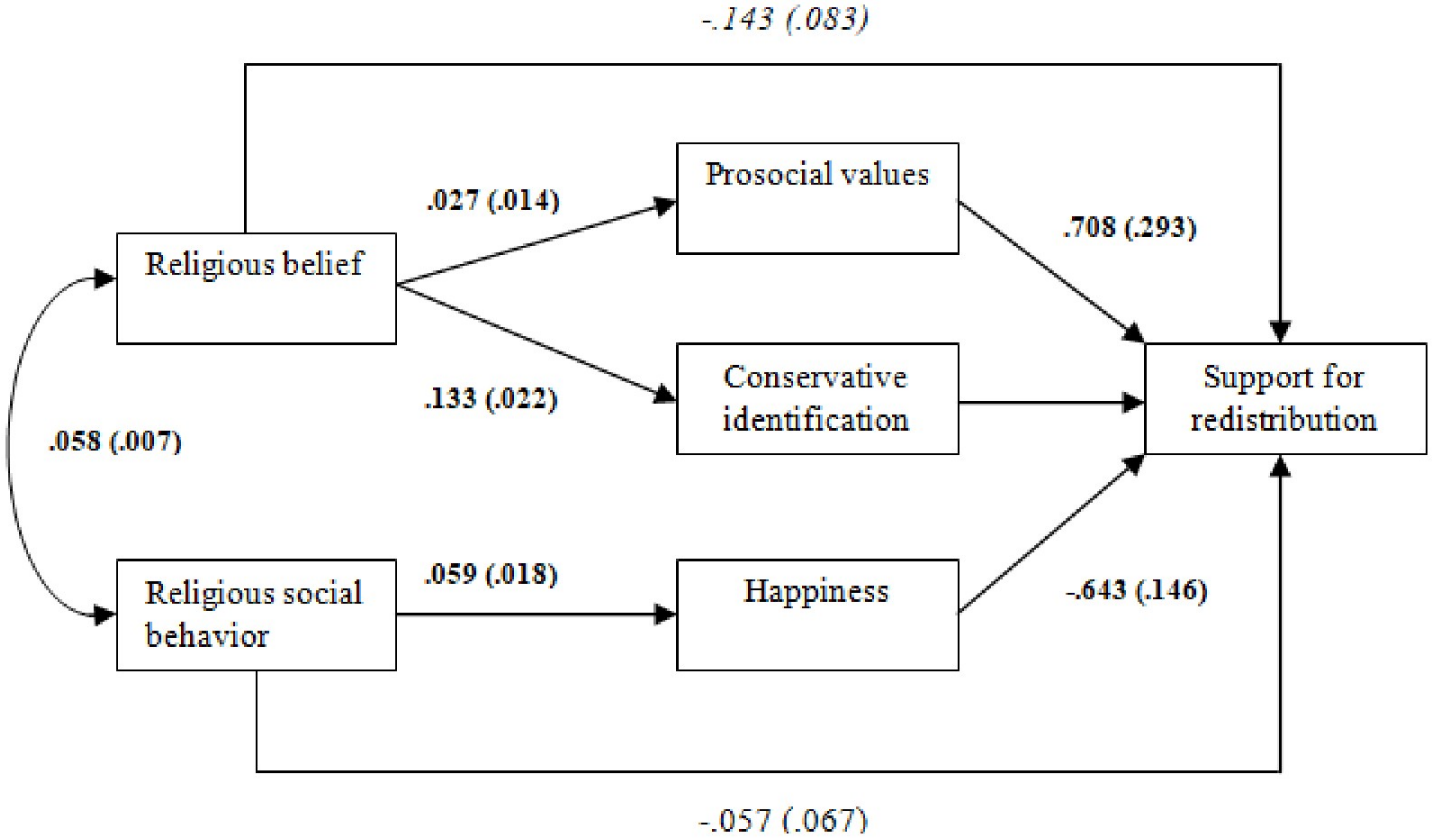
Results of Model 1.1 (within-level effects). Coefficients with standard errors in brackets. Italic entries indicate p < 0.1 (two-tailed) and bold entries indicate p < 0.05 (two-tailed).

Table 1 presents the effects of individual-level variables on support for redistribution (under “Within part of the model”) as well as the effects of the between-cluster (country level) variable (under “Between part of the model”) and Fig 3 shows within-level path coefficients and their standard errors from the most parsimonious model in Table 1 (Model 1.1).

### Model fit indices

SEM is essentially a confirmatory technique: a hypothesized model is accepted or rejected based on how well it fits the data [89]. This is why we started by inspecting the model fit indices. We choose some of the most commonly used model fit indices in the literature, motivated by Hu and Bentler’s [97] suggestion that model fit must be assessed not by looking at a single model fit indicator, but using some of these indices in combination. We chose two fit indicators that show the relative improvement in model fit compared with a statistical baseline model (null model), the comparative fit index (CFI) and Tucker-Lewis index (TLI), also known as the Non-Normed Fit Index (NNFI), and one indicator that is based on the chi-square to degrees of freedom ratio, root mean square error of approximation (RMSEA). CFI and RMSEA are among the most widely used fit indicators as they are least effected by sample size [98]. We also chose to evaluate the TLI because it is a parsimony-based index that penalizes for model complexity.

Both the CFI and TLI indicate the relative improvement in fit of the model compared with a statistical baseline model (null model), which assumes zero population covariances among the observed variables [97, 99]. CFI is a normed index that produces values that fall in-between 0 and 1, with values close to 1 representing a better fit while the TLI is non-normed in the sense that it may produce values that are slightly larger than 1 or slightly below 0 [97, 99]. While earlier studies recommended lower cut-off values, a value of CFI ≥ 0.95 and TLI ≥ 0.95 are presently recognized as indicative of good fit [97, 99].

RMSEA is scaled as a badness-of-fit index that assesses whether the model fits approximately well in the population with a value of 0 indicating the best fit [99]. Just like TLI, RMSEA is also a parsimony-adjusted index in the sense that it does not necessarily favor models with higher degrees of freedom [99]. It ıs suggested that RMSEA ≤ .05 may indicate good fit of model to the data whereas values between 0.08 and 0.10 are indicative of mediocre fit [89, 99].

Model 1.1 in Table 1, where no control variables are specified, yields fit indicators above all these thresholds (CFI = .982, TLI = .954, RMSEA = .006). Adding country-level controls (Model 1.2) increases CFI slightly while decreasing TLI and increasing RMSEA (CFI = .983, TLI = .948, RMSEA = .007). Model fit became substantively worse, however, when we added individual level control variables in Models 1.3 and 1.4. While CFI values are slightly below the accepted threshold, RMSEA and TLI are substantively worse. That is, adding demographic variables as controls to the initial model reduces rather than improves its explanatory power.

### Individual-level effects of religiosity dimensions

#### Religious belief mediators

The findings in Table 1 and Fig 3 show that the effect of religious belief on prosocial values (Path 1) is positive, as expected. That is, more devout individuals also tend to hold more prosocial values. These effects are statistically significant at the p < .05 level in three of the models (Models 1, 3, and 4). In turn, prosocial values have positive and statistically significant effects on support for redistribution (See prosocial values → DV path in Table 1 and Fig 3). These results support H1 regarding the mediating role of prosocial values. Regarding H2, in line with our expectations, religious belief has a positive effect on conservative political identification (Path 2). That is, more devout individuals tend to be more likely to identify with conservative political ideologies, which in turn decreases support for redistribution, as shown by the negative coefficients predicting the conservative ideology → dependent variable path in Table 1 and in Fig 3. Both effects are statistically different from zero in all the models.

We also consider the indirect effects of religious belief via prosocial values and conservative identification. These effects, along with their robust standard errors were calculated by the MPLUS software, and are presented in S2 Table. The indirect effects indicate the extent to which religious belief affects support for redistribution through the mediators. Although the calculated indirect effect of religious belief on support for redistribution via prosocial values for Model 1.1 is positive (coefficient = .019), it fails to reach statistical significance (p = .124) (See S2 Table). That is, although we find that religious belief positively affects prosocial values, and prosocial values positively predict support for redistribution, there is still not enough statistical evidence to support H1 that prosocial values mediate the effect of religious belief. Still, as will be discussed in detail in the next section, we find that the indirect effect of religious belief via prosocial orientations becomes statistically significant depending on the level of state welfare generosity.

In line with the predictions of H2, we find evidence for the mediating effect of conservative identification. As shown in S2 Table, the coefficient for the indirect effect of religious belief via conservative identification for Model 1.1 is calculated as -.166 with a p-value of .034. This effect is substantively larger than the indirect effect of religious belief via prosocial orientations presented above, which had a value of .019. Further, the calculated difference between the two indirect effects is -.185 with a standard error of .038, and is thus statistically significant at p < 0.000. Thus, much of the effect of religious belief on support for redistribution occurs through its effect on conservative identification. Still, the contextual analyses below show that the indirect effect of religious belief via prosocial orientations may be positive depending on welfare state generosity.

#### Religious social behavior mediators

The findings in Table 1 show that as religious social behavior increases, self-reported happiness also increases, as predicted. The results are statistically significant at the p < .00 level. Higher levels of self-reported happiness, in turn, have a negative and statistically significant effect on support for redistribution. That is, as an individual participates more in religious social activities, her levels of happiness increases, which leads to decreased support for redistribution. These effects are robust when controlling for the effects of individual and country-level control variables. The calculated indirect effect of religious social behavior is negative, as predicted by *H3* (coefficient = -.038; p = .011; See S2 Table). In addition, we find that the direct effect of religious social behavior on support for redistribution is negative but statistically not different from zero once the mediating effect of happiness is taken into account. That is, the negative effect of religious social behavior on support for redistribution is fully accounted for by its effect on levels of happiness.

### The moderating effect of state welfare generosity

The next two hypotheses, H4 and H5 predict that the effect of religious belief on prosocial values (as indicated by path 1 in Fig 2) decreases, and its effect on conservative identification (as indicated by path 2 in Fig 2) increases, as state welfare generosity increases. Using the Multilevel Structural Equation Modeling framework (M-SEM, [100, 101]), we test whether state welfare generosity, measured by the Social Security Laws Index (SSLI), has the expected effects on paths 1 and 2. M-SEM allows us to test mediation hypotheses with multilevel data and assess whether individual-level paths are moderated by level-2 variables in a single step. The equations underlying these models are presented in S1 File. We ran the model testing the moderation effects of paths 1 and 2 only due to the computational complexity of running a full model. However, the substantive results remain the same when specifying the other paths.

Note that the estimation of the moderated mediation models in Table 2 relies on a different algorithm that does not produce CFI, TLI, or RMSEA values, making it impossible to compare model fit indicators with those presented in Table 1 [89]. Instead, two model fit indicators called Akaike Information Criterion (AIC) and Bayesian Information Criterion (BIC) were estimated. Both indicators are based on the log-likelihood and model parameters, and thus both penalize models with too many parameters with BIC penalizing more harshly than AIC [88]. Unlike the other indicators, the absolute values of these indicators do not matter so there are no cut-off values indicating better fit. Instead, they can be used to compare competing models that seeks to explain the same data, with smaller values indicating better fit of the model to the data [88].

The results of the moderating effect of state welfare generosity on paths 1 and 2 are presented in Table 2. As with the previous models, we first run the most parsimonious model (Model 2.1), before adding individual and country-level control variables (Models 2.2, 2.3, 2.4). We find that state welfare generosity has a negative and statistically significant effect on path 1 in all models, supporting H4. This effect shows that the positive effect of religious belief on prosocial values weakens as state welfare provision becomes more generous. While the coefficients predicting the effect of state welfare generosity on path 2 are in the expected negative direction, they fail to achieve statistical significance.

**Fig 4:**
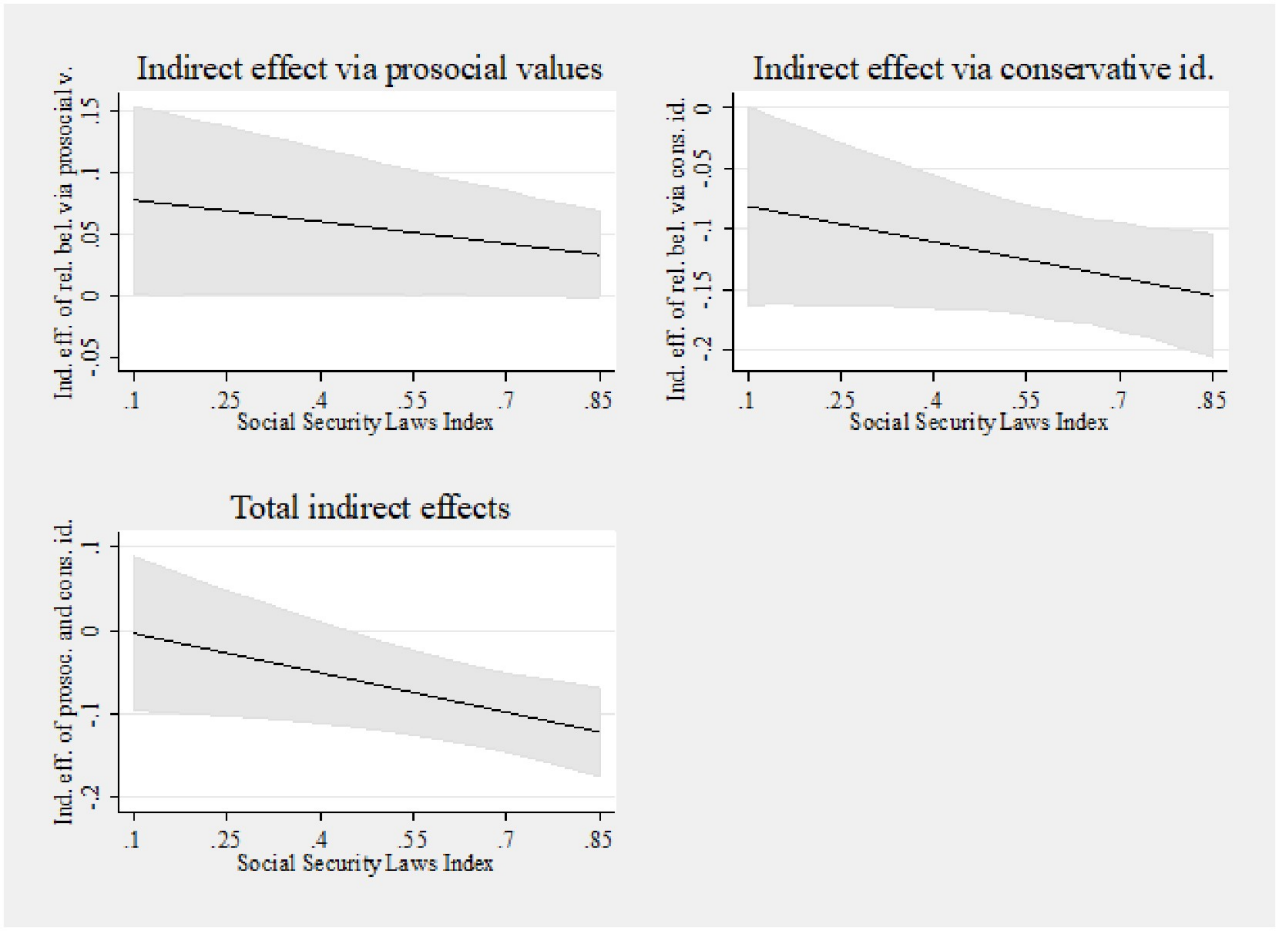
Predicted indirect effects of religious belief and religious social behavior on support for income inequality, moderated by SSLI (with 95% CIs).

For the analyses presented in Table 2, the indirect effect of religious belief via prosocial values and conservative identification is no longer a fixed value. While average indirect effects are estimated by the software (S3 Table), these effects are now moderated by the country-level welfare generosity measure and thus are conditional on the values of the Social Security Laws Index. To further investigate the indirect effects of the mediators on support for redistribution, we plotted the predicted indirect effects of religious belief via prosocial values and conservative identification, along with their confidence intervals, for different values of SSLI in Fig 4. These indirect effects and their confidence intervals were again calculated by the MPLUS software. The calculations in Fig 4 are based on the results from Model 2.1 in Table 2.

As can be seen in the upper left-hand part of Fig 4, the effect of religious belief on prosocial values decreases as state welfare generosity increases: the predicted indirect effect is calculated as 0.073 (p = .05) where welfare generosity is lowest (i.e. when SSLI is at its minimum value) and as .032 (p = .07) where welfare generosity is highest (i.e. when SSLI is at its maximum value). Thus, all else being equal, the positive indirect effect of religious belief on support for redistribution via prosocial orientations is more than twice as strong in countries with the lowest levels of state welfare generosity than in countries with the highest levels of state welfare generosity.

The upper right-hand chart in Fig 4 shows that the predicted indirect effect of religious belief on support for redistributive policies via conservative identification is in the expected direction and statistically significant for all values of the SSLI. All else being equal, the predicted indirect effect via conservative identification is lower when SSLI is higher. These results show that state welfare generosity has an important effect on the activation of psychological orientations connecting religiosity to attitudes towards redistribution. Together, these findings suggest that, when welfare provision by the state is low, religious belief contributes more to support for redistribution via its effect on prosocial orientations. Lower levels of welfare generosity also reduce the negative effect of religious belief on support for income equality via its effects on ideological orientations. Consequently, the total indirect effect of religious belief is closer to positive or null values in countries where welfare generosity is lower. This can also be seen in the lower left-hand chart in Fig 4, where the predicted total indirect effect of religious belief moves closer to zero or positive values as SSLI values decrease.

These moderating effects may explain some of the conflicting findings in the literature regarding the effect of religiosity on attitudes towards redistribution in different contexts. Thus, in countries with generous state welfare provision, such as Sweden, Switzerland, or Ukraine, the indirect effect of religious belief via prosociality is positive but smaller than in countries where state welfare generosity is low (for instance Jordan or Indonesia). Again, in contexts with high levels of welfare generosity, the negative effect of religious belief via conservative identification is stronger, thus leading to an overall negative effect of religious belief. We observe the reverse pattern for countries with low levels of state welfare generosity, such as Morocco, Malaysia, or Brazil, where the positive effect via prosocial values may override the negative effect via conservative orientations. This finding also suggests that it may be misleading to combine the belief and social behavior dimensions of religiosity in countries where state welfare generosity is low since the positive effect of religious belief and negative effect of religious social behavior may cancel each other out in these cases, leading to null results.

### Robust analysis

We conducted additional analyses to test whether the results obtained hold under a number of different circumstances. Full results and discussion are presented in the Supplementary Appendices. Below, we present information about the analyses conducted and summarize the main findings.

#### Effects across religious traditions

Our hypotheses concern the psychological mechanisms through which the belief and social behavior dimensions of religiosity affect attitudes towards redistribution. As mentioned above, we expect the hypothesized mechanisms to mostly hold across the identifiers of major religious traditions. Still, religious traditions emphasize different values to varying extents [102]. For example, Catholic beliefs arguably place more emphasis on Christian benevolence than Protestant beliefs that value individual responsibility [70]. We tested whether the proposed psychological mechanisms explaining religiosity’s effect on redistribution attitudes are similar for members of different religious traditions by running Model 1.1 (Table 1) separately for the members of the four major religious traditions in our study: Islam, Orthodox Christianity, Roman Catholicism, and Protestantism. (The number of observations for Jewish, Evangelical, and Hindu identifiers was too low to run multilevel path analyses.) S2 File presents the full results along with a more detailed discussion. Our original results generally replicate in these models, but with two major exceptions: among Protestants, religious belief has a statistically null effect on prosocial values while religious social behavior has an insignificant effect on happiness. Protestant beliefs in hard work and deservingness may explain these findings regarding the null effect of belief on prosocial orientations. A second possibility is that Protestants are divided into various denominations so that Protestant belief in hard work varies greatly among them (see, for example, [34] on Lutheran versus Calvinist views on social welfare). The diversity of Protestant denominations and congregations may also explain the null effects of religious social behavior on happiness. In fact, similar null effects of religious social behavior on institutional trust for Protestants were reported in previous studies [21].

We also test whether the proposed moderation mechanism works the same way for adherents of each major religious tradition by running the Model 2.1 in Table 2 separately for members of each tradition. Full results and discussion are presented in S2 File. The results fully support H4 and H5 for Protestant and Catholic identifiers and partially for Orthodox identifiers. Thus, we find empirical evidence that state welfare generosity moderates the effect of religious belief on prosocial orientations and conservative identification for members of three major religious traditions.

#### Testing of alternative models and reverse causality

The argument that religious belief is prior to conservative identification rests on the conceptualization of ideology as a system of “opinions, values, and beliefs about the nature of social reality that can be grouped together under some common social theme” [103]. According to this definition, ideology requires some comprehension of abstract concepts [104] and is thus expected to develop later in life than religious beliefs themselves. Research in developmental psychology suggests that comprehension of such abstract political concepts only develops in adolescence [105, 106] while some propose that it is not until the end of puberty that adolescents can grasp concepts like society, institutions, norms, and laws [107]. In contrast, religion and religious beliefs are adopted early in life, and can affect an individual’s everyday life from infancy onwards, given that most parents socialize children into their own religious beliefs and behaviours from a very young age [108]. That is, while religiosity and religious beliefs are adopted at a very young age, ideological orientations demanding more complex cognitive skills develop later in life.

Based on this developmental perspective, we expected religious belief to predict conservative orientations. Nonetheless, it is possible that conservative identification may influence levels of devoutness or involvement in religious social activities for some people at some age. We therefore tested such alternative explanations and compared our model against models that test for reverse causality between religiosity dimensions and mediators. While testing such alternative path models does not necessarily determine that the proposed model is correct, it does provide an additional statistical basis for evaluating a theory-driven model [89, 101]. Accordingly, we test a set of alternative models as to whether ideological orientations predict religious belief or religious social behavior (Models IIa and IIb in S3 File).

In addition, we tested models in which we alternated the mediators (such as religious social behavior rather than religious belief predicting prosocial orientations; see Models Ia and Ib in S3 File), and where support for redistribution is specified as a mediator (Models IIIa and IIIb in S3 File). However, all these alternative specifications yielded a poorer fit to the data than the hypothesized model. We therefore maintain that our proposed model offers the most acceptable correspondence to the data compared to alternative models [89].

#### Models utilizing different control variables

Since parsimony is one of the crucial elements of path models [91], adding too many control variables is generally not warranted. Researchers are also recommended to use control variables that are not measured with error [109]. Accordingly, path analysis research typically controls for only a few demographic variables, such as gender and/or race. As per the common practice in path analysis, we included gender, age, and level of education as controls in the models presented in Tables 1 and 2. Whereas additional potentially relevant control variables, such as union membership, income, or self-ascribed socio-economic status, can be considered, these are generally measured with a high level of error in surveys. In particular, questions on income and socioeconomic status are subject to social desirability bias, and often measured with error or yield high non-response rates [110]. In fact, this is also the case in our data since adding the income variable caused a listwise deletion of 11% of the sample (6,275 cases). However, we wanted to test whether the expected results still hold when using different controls together. The summary analysis presented in S4 File, showing that model fit is generally poorer than those in presented in Table 1 when we use different combinations of these variables as controls. Moreover, the direction and significance of path coefficients and direct and indirect effects of religiosity variables are generally robust to specifying different control variables.

In addition, it is possible that the effects of age on preferences for redistribution as well as on some mediators are not linear. Since we were interested in specifying the most parsimonious models as per the requirements for path models, we only present the linear age effects in Tables 1 and 2. We have conducted a series of robust analysis where we accounted for non-linear effects by including age and age-squared in the equations, as well as by including age group dummies. Further information and full results are presented in S5 File. We find some evidence of non-linear age effects in some but not all models and specifications. The inclusion of non-linear age effects generally decreased model fit, and more importantly did not lead to a substantive change in our major conclusions.

#### Alternative modeling strategy for moderation effects

We test H4 and H5 using an alternative approach, called the two-step strategy [75, 111]. This strategy is especially recommended to establish the robustness of the results, particularly when there are fewer than 100 level-2 observations [112, 113]. Unlike a multilevel approach in which all parameters are analyzed simultaneously, the two-step approach involves analyzing each cluster separately first, before combining and analyzing the estimates across clusters in the second step [113]. Thus, we first run the path models separately for each country and save the coefficients and standard errors of the estimates for paths 1 and 2. In the second step, we use these path coefficients as dependent variables in a linear regression using the information about the sampling errors in the dependent variable [114] to test whether state welfare generosity significantly predicts the variance in path coefficients [75, 111, 115, 116]. Detailed results and discussion of these models are presented in S6 File. All the statistically significant moderation effects established in Table 2 hold when using this approach. Using this strategy, we also test whether a range of alternative variables could explain the moderation effects, but found no empirical evidence for this. Thus, these results strengthen our findings obtained using the M-SEM approach by ruling out alternative explanations for the hypothesized moderation effects.

## Conclusions

While there is a vast literature on the individual-level determinants of welfare attitudes, the role of religion in influencing attitudes towards redistribution has only recently been addressed [7–9]. This paper contributes to the recent burgeoning literature by proposing a model of religion’s influence on support for redistribution that takes into account the multiple dimensions of religiosity and the psychological mechanisms that underlie them while simultaneously studying the moderating effect of institutional context. Whereas existing studies have generally used cases from the developed West and considered the economic attitudes of Catholic or Protestant adherents [7–9], we employ a larger comparative approach using a multilevel pooled dataset from 49 countries and various religious traditions. We also consider the effect of the prosocial dimension of religious belief on attitudes towards redistribution, which has been a relatively less studied topic.

Our findings show the nuance and complexity of the effect of religiosity on support for redistribution. In fact, our results and conclusions are in line with the recent findings that show that aggregating the different elements of individual religious experience into the broad phenomenon of “religiosity”, reducing religiosity to a single individual-level item or disregarding contextual elements, may be misleading on both theoretical and methodological grounds [23, 117, 118]. We find that the positive effect of religious belief via prosocial orientations is generally weaker compared to its negative effect via conservative orientations. Thus, considerations concerning hard work, and individualism, or government skepticism generally override the effect of religiously based prosocial orientations. However, we also show that prosocial considerations may exert greater influence on attitudes towards redistribution particularly when states provide less generous welfare benefits. While we do not present a direct test of the mechanism underlying this effect, we suggest that religiously-motivated welfare provision becomes more influential in when states provide less generous welfare benefits, and reinforces the relationship between religious beliefs and prosocial orientations. This finding suggests that the role of religious belief in supporting redistributive polices may actually be positive in contexts outside the most often studied Western cases that have high levels of welfare generosity. Such positive effects have indeed been reported for Morocco, Indonesia, and Pakistan [20], where state welfare generosity is low, whereas the effect of religious belief on support for redistribution in the West tends to be mostly negative [7, 9]. These findings highlight the importance of considering context in studying religiosity and redistribution attitudes as the competing considerations underlying belief’s effect lead to different results in different institutional settings.

Prosocial orientations provide a link between religious beliefs and support for redistribution, but their effect on redistribution attitudes weaken as states provide more generous welfare services. Thus, we may expect more resistance to religiously-based discourses on helping and benevolence in contexts where welfare generosity is high, as in the case of the conservative backlash against Pope Francis’s support for efforts to solve problems of inequality and poverty [119]. Nevertheless, it may still be possible for religious or political leadership to connect religious beliefs to support for more income equality by continuously stressing the importance of caring for others. In particular, times of economic hardship may lead to situations where state welfare provision may be inadequate could make religiously-based prosociality messages more effective in garnering public support for more government action.

In addition, we find that religious social behavior leads to lower levels of support for redistribution via happiness. However, the effect of the social behavior dimension on support for redistribution is always negative. Therefore, in countries where the effect of religious belief is positive due to low state welfare generosity, pooling different dimensions of religiosity may lead to biased findings. This shows the importance of distinguishing between different dimensions of religiosity, as in different settings, the effect of belief on attitudes towards redistribution may be positive, and merging the two dimensions in a single measure may lead to null findings.

While our approach offers a way of reconciling some of the conflicting findings in the literature, it also has a number of limitations. First, while our large-n approach allows us to test the effects of country-level variables on individual attitudes, we are also restricted by existing datasets in some respects. As with all cross-national surveys, the WVS does not collect detailed data on denominational, congregational, or sectarian affiliation for a large number of countries, which prevents researchers from conducting detailed analyses of the effect of the belonging dimension. Thus we were unable to examine the extent to which the hypothesized mechanisms varied across congregations or denominations within major religious traditions. Future studies could collect detailed information at the congregation or denomination level to conduct more detailed analysis of the role of the belonging dimension. Next, we used common proxy measures and short scales for some of the variables, including religious belief and happiness due to lack of detailed survey batteries. In the future, researchers can improve the internal validity of their designs by using more fine-tuned measures.

Second, our analysis does not claim to explain the full variance in attitudes towards redistribution, and there are elements within religion that were unattended due to lack of information in the dataset. For example, although we found that the proposed psychological mechanisms largely held across members of major religious traditions, some unexplained differences in the strength of relationships remained, which can be the subject of future works. Third, we do acknowledge that the relationship between religion and welfare could be unique in several respects in different countries and at different time points. Our study could not encompass the full contextual and temporal variance given the large-N approach, but researchers choosing to utilize case studies could provide answers with regards to these questions.

Needless to say, proposing that religiosity and its components are associated with certain psychological orientations does not deny the distinctiveness of religiosity as a belief and value system. Rather, our work contributes to the existing literature by acknowledging religion’s complexity and its interconnectedness with both individual- and system-level elements, such as welfare arrangements. We thus believe that the fact that a section of the effect of religiosity is due to its effect on the devout’s psyche, and the highly complex and partial nature of these effects does not deny the uniqueness of religion as a phenomenon.

## Supporting information

S1 Table Variables, data sources, and summary statistics. (DOCX)

S2 Table Direct and indirect effects of religious belief and religious social behavior (Models 1.1–1.4). (DOCX)

S3 Table Direct and indirect effects of religious belief moderated by SSL index (Models 2.1–2.4). (DOCX)

S1 File Equations underlying Models 1.1–1.4 and 2.1–2.4. (DOCX)

S2 File Mediation and moderation effects across religious traditions. (DOCX)

S3 File Testing of alternative models and reverse causality. (DOCX)

S4 File Models utilizing different control variables. (DOCX)

S5 File Robust analysis of age effects. (DOCX)

S6 File Alternative modeling strategy to test for moderation effects. (DOCX)

## References

[1] Davidson JD, Pyle RE. Public Religion and Economic Inequality In: Swatos WJ, Wellman JJ, editors. The Power of Religious Publics. Westport, CT: Praeger; 1999 p. 101–14.

[2] Hart S. The cultural dimension of social movements: A theoretical reassessment and literature review. Sociol Relig. 1996;57(1):87–100. 10.2307/3712006

[3] Benabou R, Tirole J. Belief in a just world and redistributive politics. Q J Econ. 2006;121(2):699–746.

[4] Clark A, Lelkes O. Deliver Us from Evil: Religion as Insurance. 2005.

[5] Dehejia R, DeLeire T, Luttmer EFP. Insuring consumption and happiness through religious organizations. J Public Econ. 2007;91(1–2):259–79.

[6] Huber JD, Stanig P. Church-state separation and redistribution. J Public Econ. 2011;95(7–8):828–36.

[7] Jordan J. Religious Belief, Religious Denomination, and Preferences for Redistribution: A Comparison across 13 Countries. West Eur Polit. 2014;37(1):19–41.

[8] Stegmueller D, Scheepers P, Rossteutscher S, de Jong E. Support for Redistribution in Western Europe: Assessing the role of religion. Eur Sociol Rev. 2012;28(4):482–97. 10.1093/esr/jcr011

[9] Stegmueller D. Religion and Redistributive Voting in Western Europe. J Polit. 2013;75(4):1064–76. 10.1017/S0022381613001023

[10] Be’ery G, Ben-Nun Bloom P. God and the Welfare State—Substitutes or Complements? An Experimental Test of the Effect of Belief in God’s Control. Plos One. 2015;10(6). 10.1371/journal.pone.0128858 PMC4463850 26061050

[11] Bloom P. Religion, Morality, Evolution. Annu Rev Psychol. 2012;63:179–99. 10.1146/annurev-psych-120710-100334 21943167

[12] Putnam RD, and Campbell D.E. American Grace: How Religion Divides and Unites Us. New York: Simon & Schuster; 2010.

[13] Malka A, Soto CJ, Cohen AB, Miller DT. Religiosity and Social Welfare: Competing Influences of Cultural Conservatism and Prosocial Value Orientation. J Pers. 2011;79(4):763–92. 10.1111/j.1467-6494.2011.00705.x 21682729

[14] Smidt CE, Kellstedt LA, Guth JL. The Role of Religion in American Politics: Explanatory Theories and Associated Analytical and Measurement Issues In: Smidt Corwin K LA, and Guth James L., editor. Oxford Handbook on Religion and American Politics. Oxford: Oxford University Press; 2009 p. 3–42.

[15] Wald KD, Wilcox C. Getting religion: Has political science rediscovered the faith factor? Am Polit Sci Rev. 2006;100(4):523–9.

[16] Arikan G. Values, Religiosity and Support for Redistribution and Social Policy in Turkey. Turk Stud. 2013;14(1):34–52. 10.1080/14683849.2013.766980

[17] Davis NJ, Robinson RV. Claiming Society for God: Religious Movements and Social Welfare. Bloomington, IN: Indiana University Press; 2012.

[18] De La O AL, Rodden JA. Does religion distract the poor? Income and issue voting around the world. Comp Polit Stud. 2008;41(4–5):437–76.

[19] Guiso L, Sapienza P, Zingales L. People’s opium? Religion and economic attitudes. J Monetary Econ. 2003;50(1):225–82.

[20] Pepinsky TB, Welborne BC. Piety and Redistributive Preferences in the Muslim World. Polit Res Quart. 2011;64(3):491–505. 10.1177/1065912909359404

[21] Ben-Nun Bloom P, Arikan G. Religion and Support for Democracy: A Cross-National Test of the Mediating Mechanisms. Brit J Polit Sci. 2013;43:375–97. 10.1017/S0007123412000427

[22] Dekker P, Halman L. The Values of Volunteering: Cross-Cultural Perspectives. New York: Kluwer Academic Publishers; 2003.

[23] Ben-Nun Bloom P, Arikan G, Courtemanche M. Religious Social Identity, Religious Belief, and Anti-Immigration Sentiment. Am Polit Sci Rev. 2015;109(2):203–21. 10.1017/S0003055415000143

[24] Kotler-Berkowitz LA. Religion and voting behaviour in Great Britain: A reassessment. Brit J Polit Sci. 2001;31:523–54.

[25] Hoffman M, Jamal A. Religion in the Arab Spring: Between Two Competing Narratives. J Polit. 2014;76(3):593–606.

[26] Kilburn HW. Religion and foundations of American public opinion towards global climate change. Environ Polit. 2014;23(3):473–89.

[27] Philpott D. Explaining the political ambivalence of religion. Am Polit Sci Rev. 2007;101(3):505–25.

[28] Ben-Nun Bloom P, Arikan G, Lahav G. The effect of perceived cultural and material threats on ethnic preferences in immigration attitudes. Ethnic Racial Stud. 2015;38(10):1760–78. 10.1080/01419870.2015.1015581

[29] Randolph-Seng B, and Nielsen Michael E. Honesty: One Effect of Primed Religious Representations. International Journal for the Psychology of Religion 2007;17(4):303–15.

[30] Saroglou V, Delpierre V, Dernelle R. Values and religiosity: a meta-analysis of studies using Schwartz’s model. Pers Indiv Differ. 2004;37(4):721–34.

[31] Shariff AF, Norenzayan A. God is watching you—Priming god concepts increases prosocial behavior in an anonymous economic game. Psychol Sci. 2007;18(9):803–9. 10.1111/j.1467-9280.2007.01983.x 17760777

[32] Safley TM. The Reformation of Charity: The Secular and the Religious in Early Modern Poor Relief. Safley TM, editor. Boston: Brill Academic Publishers; 2003.

[33] Trattner WI. From Poor Law to Welfare State: A History of Social Welfare in America. Sixth ed New York: The Free Press; 1998.

[34] Van Kersbergen K, Manow P. Religion, Class Coalitions, and Welfare States. Cambridge: Cambridge University Press; 2009.

[35] Brooks AC. Who really cares: The surprising truth about compassionate conservatism. New York: Basic Books; 2006.

[36] Schwartz SH, Huismans S. Value Priorities and Religiosity in 4 Western Religions. Soc Psychol Quart. 1995;58(2):88–107.

[37] Blouin DD, Robinson RV, Starks B. Are Religious People More Compassionate and Does This Matter Politically? Polit Relig. 2013;6(3):618–45.

[38] Norris P, Inglehart R. Sacred and Secular: Religion and Politics Worldwide. New York: Cambridge University Press; 2004.

[39] Abu-Saad I. Individualism and islamic work beliefs. J Cross Cult Psychol. 1998;29(2):377–83.

[40] Lee W, and Roemer John. Values and Politics in the U.S.: An Equilibrium Analysis of the 2004 Election. 2005.

[41] Jaeger MM. Does left-right orientation have a causal effect on support for redistribution causal analysis with cross-sectional data using instrumental variables. Int J Public Opin R. 2008;20(3):363–74.

[42] Frank T. Whats’ the Matter with Kansas? New York: Metropolitan Books; 2005

[43] Scheve K, Stasavage D. Religion and preferences for social insurance. Q J Polit Sci. 2006;1(3):255–86.

[44] DeLeire T, Kalil A. Does Consumption Buy Happiness? Evidence from the United States. International Review of Economics. 2010;57(2):163–76. https://link.springer.com/journal/volumesAndIssues/12232.

[45] Chaves M. Congregations in America. Cambridge, MA: Harvard University Press; 2004.

[46] Greenberg J, Pyszczynski Tom, and Solomon Sheldon. The Causes and Consequences of a Need for Self-Esteem: A Terror Management Theory In: Baumeister RF, editor. Public Self and Private Self. New York: Springer-Verlag; 1986 p. 189–212.

[47] Jonas E, Fischer P. Terror management and religion: Evidence that intrinsic religiousness mitigates worldview defense following mortality salience. J Pers Soc Psychol. 2006;91(3):553–67. 10.1037/0022-3514.91.3.553 16938037

[48] Vail KE, Rothschild ZK, Weise DR, Solomon S, Pyszczynski T, Greenberg J. A Terror Management Analysis of the Psychological Functions of Religion. Pers Soc Psychol Rev. 2010;14(1):84–94. 10.1177/1088868309351165 19940284

[49] Ellison CG, Fan D. Daily spiritual experiences and psychological well-being among US adults. Soc Indic Res. 2008;88(2):247–71.

[50] Van Cappellen P, Toth-Gauthier M, Saroglou V, Fredrickson BL. Religion and Well-Being: The Mediating Role of Positive Emotions. J Happiness Stud. 2016;17(2):485–505. 10.1007/s10902-014-9605-5

[51] Vishkin A, Ben-Nun Bloom P, Tamir M. Always Look on the Bright Side of Life: Religiosity, Emotion Regulation and Well-Being in a Jewish and Christian Sample. J Happiness Stud. 2018.

[52] Clore GL, Huntsinger JR. How emotions inform judgment and regulate thought. Trends Cogn Sci. 2007;11(9):393–9. 10.1016/j.tics.2007.08.005 17698405 PMC2483304

[53] Forgas JP, Bower GH. Mood Effects on Person-Perception Judgments. J Pers Soc Psychol. 1987;53(1):53–60. 10.1037/0022-3514.53.1.53 3612493

[54] Cohn MA, Fredrickson BL, Brown SL, Mikels JA, Conway AM. Happiness Unpacked: Positive Emotions Increase Life Satisfaction by Building Resilience. Emotion. 2009;9(3):361–8. 10.1037/a0015952 19485613 PMC3126102

[55] Lyubomirsky S, King L, Diener E. The benefits of frequent positive affect: Does happiness lead to success? Psychol Bull. 2005;131(6):803–55. 10.1037/0033-2909.131.6.803 16351326

[56] Headey BaRV. Does Happiness Induce a Rosy Outlook? In: Veenhoven R, editor. How harmfull is happiness? Consequences of enjoying life or not. The Netherlands: Universitaire Pers Rotterdam; 1989 p. 106–27

[57] Hudson J. Institutional trust and subjective well-being across the EU. Kyklos. 2006;59(1):43–62.

[58] Listhaug O. Confidence in Institutions—Findings from the Norwegian Values Study. Acta Sociol. 1984;27(2):111–22. 10.1177/000169938402700202

[59] Graham CL, Pettinato S. Happiness and Hardship: Opportunity and Insecurity in New Market Economies: Brookings Institution Press; 2001.

[60] Blekesaune M. Economic conditions and public attitudes to welfare policies. Eur Sociol Rev. 2007;23(3):393–403.

[61] Hasenfeld Y, Rafferty JA. The Determinants of Public-Attitudes toward the Welfare-State. Soc Forces. 1989;67(4):1027–48.

[62] Alesina A, Di Tella R, MacCulloch R. Inequality and happiness: are Europeans and Americans different? J Public Econ. 2004;88(9–10):2009–42. 10.1016/j.jpubeco.2003.07.006

[63] Gaviria A, Graham C, Braido LHB. Social Mobility and Preferences for Redistribution in Latin America Economía. 2007;8(1):55–96.

[64] Kellstedt LA, Green JC, Guth JL, Smidt CE. Is There a Culture War? Religion and the 1996 Election. Annual Meeting of the American Political Science Association; Washington, D.C.1997.

[65] Wald KD, Smidt CE. Measurement Strategies in the Study of Religion and Politics In: Leege DC, Kellstedt LA, editors. Rediscovering the Religious Factor in American Politics. New York: M.E. Sharpe; 1993 p. 26–49.

[66] Diener E, Tay L, Myers DG. The Religion Paradox: If Religion Makes People Happy, Why Are So Many Dropping Out? J Pers Soc Psychol. 2011;101(6):1278–90. 10.1037/a0024402 21806304

[67] Svallfors S. Worlds of welfare and attitudes to redistribution: A comparison of eight western nations. Eur Sociol Rev. 1997;13(3):283–304. 10.1093/oxfordjournals.esr.a018219

[68] Gill A, Lundsgaarde E. State welfare spending and religiosity—A cross-national analysis. Ration Soc. 2004;16(4):399–436.

[69] Karakoc E, Baskan B. Religion in Politics: How Does Inequality Affect Public Secularization? Comp Polit Stud. 2012;45(12):1510–41.

[70] Kahl S. Religious Doctrines and Poor Relief: A Different Causal Pathway In: Manow KvKaP, editor. Religion, Class Coalitions, and Welfare States. Cambridge: Cambridge University Press; 2009 p. 267–96.

[71] Zehavi A. The faith-based initiative in comparative perspective—Making use of religious providers in Britain and the United States. Comp Polit. 2008;40(3):331-+.

[72] Jawad R. Religion and Faith-Based Welfare. Bristol: The Policy Press; 2012.

[73] Franko W, Tolbert CJ, Witko C. Inequality, Self-Interest, and Public Support for "Robin Hood" Tax Policies. Polit Res Quart. 2013;66(4):923–37.

[74] Bartels L. Homer Gets a Tax Cut: Inequality and Public Policy in the American Mind. Perspectives on Politics 2005;3(1):15–31.

[75] Gingrich J. Visibility, Values, and Voters: The Informational Role of the Welfare State. J Polit. 2014;76(2):565–80.

[76] Blofield M, Luna JP. Public Opinion and Income Inequalities in Latin America In: Blofield M, editor. The Great Gap: Inequality and the Politics of Redistribution in Latin America. University Park: Penn State Press; 2011 p. 147–81.

[77] Gaeta GL. In the Mood for Redistribution: An Empirical Analysis of Individual Preferences for Redistribution in Italy. MPRA Paper2011.

[78] Jakobsen TG. Welfare Attitudes and Social Expenditure: Do Regimes Shape Public Opinion? Soc Indic Res. 2011;101(3):323–40.

[79] Klor EF, Shayo M. Social identity and preferences over redistribution. J Public Econ. 2010;94(3–4):269–78.

[80] Shayo M. A Model of Social Identity with an Application to Political Economy: Nation, Class, and Redistribution. Am Polit Sci Rev. 2009;103(2):147–74.

[81] Ben-Nun Bloom P, Arikan G. A Two-edged Sword: The Differential Effect of Religious Belief and Religious Social Context on Attitudes towards Democracy. Polit Behav. 2012;34(2):249–76. 10.1007/s11109-011-9157-x

[82] Schwartz SH. Universals in the Content and Structure of Values: Theory and Empirical Tests in 20 Countries In: Zanna M, editor. Advances in Experimental Social Psychology. New York: Academic Press; 1992 p. 1–65.

[83] Schwartz SH. A Theory of Cultural Value Orientations: Explication and Applications. Comparative Sociology. 2006;5(2–3):137–82.

[84] Schwartz SH. Value orientations: Measurement, antecedents and consequences across nations In: Roberts JR, Fitzgerald R, Eva G, editors. Measuring attitudes cross-nationally: Lessons from the European Social Survey. London: Sage; 2006 p. 169–203.

[85] Feldman S. Enforcing social conformity: A theory of authoritarianism. Polit Psychol. 2003;24(1):41–74.

[86] Schiefer D. Cultural Values and Group-Related Attitudes: A Comparison of Individuals With and Without Migration Background Across 24 Countries. J Cross Cult Psychol. 2013;44(2):245–62. 10.1177/0022022112444898

[87] Botero JC, Djankov S, La Porta R, Lopez-de-Silanes F, Shleifer A. The regulation of labor. Q J Econ. 2004;119(4):1339–82. 10.1162/0033553042476215

[88] Kaplan D. Structural Equation Modeling: Foundations and Extensions. Second ed California: Sage; 2009.

[89] Kline RB. Principles and Practice of Structural Equation Modeling. Third ed New York: Guilford Press; 2011.

[90] Bollen KA, Rabe‐Hesketh S, Skrondal A. Structural Equation Models In: Collier HABaD, editor. The Oxford Handbook of Political Methodology. New York: Oxford University Press; 2008 p. 432–55.

[91] Loehlin JC. Latent variable models: An introduction to factor, pathm and structural analysis. New Jersey: Lawrence Erlbaum Associates; 1987.

[92] Canetti-Nisim D. The effect of religiosity on endorsement of democratic values: The mediating influence of authoritarianism. Polit Behav. 2004;26(4):377–98. 10.1007/s11109-004-0901-3

[93] Bowen N, Guo S. Structural Equation Modeling. New York: Oxford University Press; 2011.

[94] Perry JL, Brudney JL, Coursey D, Littlepage L. What drives morally committed citizens? A study of the antecedents of public service motivation. Public Admin Rev. 2008;68(3):445–58.

[95] White H. A Heteroskedasticity-Consistent Covariance-Matrix Estimator and a Direct Test for Heteroskedasticity. Econometrica. 1980;48(4):817–38. 10.2307/1912934

[96] Muthen LK, Muthen BO. MPLUS User’s Guide. Eighth ed Los Angeles, CA 2017.

[97] Hu LT, Bentler PM. Cutoff Criteria for Fit Indexes in Covariance Structure Analysis: Conventional Criteria Versus New Alternatives. Struct Equ Modeling. 1999;6(1):1–55. 10.1080/10705519909540118

[98] Kenny DA, McCoach DB. Effect of the number of variables on measures of fit in structural equation modeling. Structural Equation Modeling. 2003;10(3):333–51. 10.1207/S15328007sem1003_1

[99] Kaplan D, Elliott PR. A Didactic Example of Multilevel Structural Equation Modeling Applicable to the Study of Organizations. Struct Equ Modeling. 1997;4(1):1–24. 10.1080/10705519709540056

[100] Preacher KJ, Zhang Z, Zyphur MJ. Alternative Methods for Assessing Mediation in Multilevel Data: The Advantages of Multilevel SEM. Struct Equ Modeling. 2011;18(2):161–82. 10.1080/10705511.2011.557329

[101] Preacher KJ, Zyphur MJ, Zhang Z. A General Multilevel SEM Framework for Assessing Multilevel Mediation. Psychol Methods. 2010;15(3):209–33. 10.1037/a0020141 20822249

[102] Hoover DR, Martinez MD, Reimer SH, Wald KD. Evangelicalism meets the continental divide: Moral and economic conservatism in the United States and Canada. Polit Res Quart. 2002;55(2):351–74.

[103] Federico C. The Structure, Foundations, and Expression of Ideology In: Berinsky A, editor. New Directions in Public Opinion Second ed New York: Routledge; 2015 p. 81–103.

[104] Freeden M. Ideologies and Political Theory: A Conceptual Approach. New York: Clarendon Press; 1996.

[105] Piaget J, Weil AM. The Development in Children of the Idea of Homeland and Relations with Other Countries. International Social Science Bulletin 1951;3:561–78.

[106] Sigel IE, Cocking RE. Cognitive Development from Childhood to Adolescence: A Constructivist Perspective. New York: Holt, Rinehart and Winston; 1977.

[107] Torney-Purta J. Youth in Relation to Social Institutes In: Feldman aGRE S.S., editor. At the Threshold: The Developing Adolescent. Cambridge: Harvard University Press; 1990 p. 457–77.

[108] Myers SM. An interactive model of religiosity inheritance: The importance of family context. Am Sociol Rev. 1996;61(5):858–66. 10.2307/2096457

[109] Williams LJ, Vandenberg RJ, Edwards JR. Structural Equation Modeling in Management Research: A Guide for Improved Analysis. Acad Manag Ann. 2009;3:543–604.

[110] Tourangeau R, Yan T. Sensitive questions in surveys. Psychol Bull. 2007;133(5):859–83. 10.1037/0033-2909.133.5.859 17723033

[111] Duch RM, Stevenson RT. The Economic Vote: How Political and Economic Institutions Condition Election Results. Cambridge: Cambridge University Press; 2008.

[112] Bowers J, Drake KW. EDA for HLM: Visualization when probabilistic inference fails. Polit Anal. 2005;13(4):301–26. 10.1093/pan/mpi031

[113] Gelman A. Two-stage regression and multilevel modeling: A commentary. Polit Anal. 2005;13(4):459–61.

[114] Lewis JB, Linzer DA. Estimating regression models in which the dependent variable is based on estimates. Polit Anal. 2005;13(4):345–64. 10.1093/pan/mpi026

[115] Huber JD, Kernell G, Leoni EL. Institutional context, cognitive resources and party attachments across democracies (vol 13, pg 365, 2006). Polit Anal. 2006;14(4).

[116] Jusko KL, Shively WP. Applying a two-step strategy to the analysis of cross-national public opinion data. Polit Anal. 2005;13(4):327–44.

[117] Arikan G, Ben-Nun Bloom P. Social Values and Cross-National Differences in Attitudes towards Welfare. Polit Stud-London. 2015;63(2):431–48. 10.1111/1467-9248.12100

[118] Hoffman M. Communal Religion, Secterian Interests, and Democracy. Princeton, NJ: Princeton University; 2016.

[119] McElroy R. Market Assumptions: Pope Francis’ Challenge to Economic Inequality. America Magazine 2014 3 11.

