# Correction: "I was hungry and you gave me food": Religiosity and attitudes toward redistribution

**DOI:** 10.1371/journal.pone.0215935

**Published:** 2019-04-18

**Authors:** The PLOS ONE Staff

There are errors in the Funding statement. The publisher apologizes for the errors. The correct Funding statement is as follows: This research was supported by grants from Turkish Academy of Sciences Young Scientist Award Program (Türkiye Bilimler Akademisi GEBIP2015; Dr. Gizem Arikan) and Israeli Science Foundation (ISF676/13, Dr. Pazit Ben-Nun Bloom).

## References

[1] Arikan G, Ben-Nun Bloom P (2019) “I was hungry and you gave me food”: Religiosity and attitudes toward redistribution. PLoS ONE 14(3): e0214054 10.1371/journal.pone.0214054 30901376 PMC6430507

