# Supplementary material for: “I was hungry and you gave me food”: Religiosity and attitudes toward redistribution
Source: PLoS One. 2019 Mar 22;14(3):e0214054. doi: 10.1371/journal.pone.0214054 (PMC6430507; doi:10.1371/journal.pone.0214054)
Supplement: S1 Table — (DOCX) [file pone.0214054.s001.docx]

# S1 Table. Variables, Data Sources, and Summary Statistics*

| **Variables** | **Source, Operationalization, and Summary Statistics (number of observations / mean / standard deviation / minimum value / maximum value)** |
| --- | --- |
| *Dependent variable* |  |
| Support for income equality | World Values Surveys, Wave 5. “Now I’d like you to tell me your views on various issues. How would you place your views on this scale? 1 means you agree completely with the statement on the left; 10 means you agree completely with the statement on the right; and if your views fall somewhere in between, you can choose any number in between.” “Incomes should be made more equal” (1) and “We need larger income differences as incentives for individual effort” (10). (Recoded so that higher values represent greater support for income equality.)  n=63882 / x̅ =5.1 / s=2.93 / min=1 / max=10 |
| *Individual-level variables* |  |
| Religious belief | World Values Surveys, Wave 5. Summary index of two items: “Independently of whether you attend religious services or not, would you say you are: A religious person / Not a religious person / A convinced atheist” (Religious is coded 1, and the other options are coded 0); “How important is God in your life? Please use this scale to indicate. 10 means “very important” and 1 means “not at all important.” (rescaled to vary between 0-1) The additive index was constructed by giving equal weight to both items after they were recoded, and the final version of the index was rescaled to vary between 0 and 1. n=62709 / x̅ =.75 / s=.32 / min=0 / max=1 |
| Religious social behavior | World Values Surveys, Wave 5. Summary index of two items; each item carries equal weight after recoding: “Apart from weddings and funerals, about how often do you attend religious services these days?” (7-point scale, recoded to vary between 0 and 1 where 1 indicates the most frequent attendance); “Now I am going to read off a list of voluntary organizations. For each one, could you tell me whether you are an active member, an inactive member, or not a member of that type of organization?” “Church or religious organization” (Not a member = 0 / active member = 1 / inactive member = 0). The final index was rescaled to vary between 0 and 1. n=59490 / x̅ =.48 / s=.37 / min=0 / max=1 |
| Prosocial values | World Values Surveys, Wave 5. Shortened version of Schwartz PVQ full wording: “Now I will briefly describe some people. Using this card, would you please indicate for each description whether that person is very much like you, like you, somewhat like you, not like you, or not at all like you?”  Two items that tap self-enhancement values (benevolence and universalism) were added after the centering procedure described by Schwartz (2006, 2012): “It is important to this person to help the people nearby and to care for their well-being,” and “Looking after the environment and caring for nature are important to this person.” Three values representing the opposite higher-order dimension self-enhancement (power, achievement, hedonism) were added after the centering procedure described by Schwartz (2006, 2012): “It is important to this person to be rich, or to have a lot of money and expensive things,” “Being very successful is important to this person, along with being recognized for his/her achievements,” “It is important to this person to have a good time; to spoil oneself.”  Each respondent’s score on self-direction values are subtracted from self-transcendence values, such that the final measure to captures the relative weight respondents place on self-transcendence over self-enhancement. (See Schwartz 2006) The final measure is rescaled to vary between 0 and 1. n=54088 / x̅ =.60 / s=.13 / min=0 / max=1 |
| Conservative identification | World Values Surveys, Wave 5. “In political matters, people talk of ‘the left’ and ‘the right.’ How would you place your views on this scale, generally speaking?” (1 Left; 10 Right) Rescaled to vary between 0 -1. n=49569 / x̅ =.52 / s=.26 / min=0 / max=1 |
| Happiness | World Values Surveys, Wave 5. “Taking all things together, would you say you are…” Very happy / Quite happy / Not very happy / Not at all happy. Rescaled to vary between 0 -1. n=65336 / x̅ =.7 / s=.25 / min=0 / max=1 |
| Age | World Values Surveys, Wave 5. Age of respondent.  n=65747 / x̅=41.95 / s=16.79 / min=15 / max=98 |
| Male | World Values Surveys, Wave 5. Gender of respondent, coded 1 if respondent is male, and 0 if female.  n=65928 / x̅=.48 / s=.49 / min=0 / max=1 |
| Low education (dummy) | World Values Surveys, Wave 5. “What is the highest educational level that you have attained?” The response options slightly differed in each country, and the responses were coded into eight general categories by the WVS team. We coded all those who fall under the categories “inadequately completed elementary education”, “completed elementary education”, and “incomplete secondary school” as low levels of education.  n= 65502 / x̅=.35 / s=.47 / min=0 / max=1 |
| *Level-2 variables* |  |
| State welfare generosity | Botero et al. 2004. Social Security Laws Index: Measures social security benefits as the average of three indices developed by researchers: (1) old age, disability and death benefits; (2) sickness and health benefits; and (3) unemployment benefits. n=42 / x̅ =.6 / s=.23 / min=.1 / max=.85 |
|  |  |
| GDP per capita (PPP) | World Bank; survey year. Logged. n=46 / x̅ =9.3 / s=1.15 / min=6.67 / max=10.92 |
| Religious fractionalization | Alesina et al. 2003. n=48 / x̅ =.46 / s=.25 / min=.004 / max=.86 |
| *Countries included* | Andorra, Argentina, Australia, Brazil, Bulgaria, Burkina Faso, Canada, Chile, Colombia, Cyprus, Ethiopia, Finland, France, Georgia, Germany, Ghana, Great Britain, Guatemala, Hong Kong, India, Indonesia, Italy, Japan, Jordan, Malaysia, Mali, Mexico, Moldova, Morocco, Netherlands, New Zealand, Norway, Peru, Poland, Romania, Serbia, Slovenia, South Africa, South Korea, Spain, Sweden, Switzerland, Taiwan, Trinidad, Turkey, Ukraine, United States, Uruguay, Zambia |

* All individual-level variables, with the exception of age, were recoded to vary between 0 and 1 to facilitate interpretation.
