# Supplementary material for: “I was hungry and you gave me food”: Religiosity and attitudes toward redistribution
Source: PLoS One. 2019 Mar 22;14(3):e0214054. doi: 10.1371/journal.pone.0214054 (PMC6430507; doi:10.1371/journal.pone.0214054)
Supplement: S2 Table — (DOCX) [file pone.0214054.s002.docx]

# S2 Table. Direct and Indirect Effects of Religious Belief and Religious Social Behavior (Models 1.1 – 1.4)

|  | **Model 1.1** | **Model 1.2** | **Model 1.3** | **Model 1.4** |
| --- | --- | --- | --- | --- |
| Total effect of religious belief | **-.290 (.085)** | **-.320 (.088)** | **-.381 (.086)** | **-.409 (.088)** |
| Total indirect effect of religious belief | **-.147 (.035)** | **-.164 (.033)** | **-.158 (.034)** | **-.174 (.032)** |
| Indirect effect via prosocial values | .019 (.012) | .019 (.014) | .018 (.011) | .019 (.013) |
| Indirect effect via conservative identification | **-.166 (.034)** | **-.183 (.032)** | **-.176 (.034)** | **-.194 (.031)** |
| Direct effect of religious belief | *-.143 (.083)* | *-.156 (.093)* | **-.223 (.082)** | **-.234 (.091)** |
|  |  |  |  |  |
| Total effect of religious social behavior | -.095 (.066) | -.083 (.071) | -.100 (.064) | -.085 (.068) |
| Total indirect effect of religious social behavior | **-.038 (.015)** | **-.038 (.017)** | **-.035 (.013)** | **-.034 (.015)** |
| Indirect effect via happiness | **-.038 (.015)** | **-.038 (.017)** | **-.035 (.013)** | **-.034 (.015)** |
| Direct effect of religious social behavior | -.046 (.072) | -.046 (.072) | -.065 (.064) | -.051 (.069) |
|  |  |  |  |  |
| N1/N2 | 65980 / 49 | 55681 / 40 | 65278 / 49 | 55028 / 40 |
