# Supplementary material for: “I was hungry and you gave me food”: Religiosity and attitudes toward redistribution
Source: PLoS One. 2019 Mar 22;14(3):e0214054. doi: 10.1371/journal.pone.0214054 (PMC6430507; doi:10.1371/journal.pone.0214054)
Supplement: S3 Table — (DOCX) [file pone.0214054.s003.docx]

# S3 Table. Direct and Indirect Effects of Religious Belief Moderated by SSL Index (Models 2.1 – 2.4)

|  | **Model 2.1** | **Model 2.2** | **Model 2.3** | **Model 2.4** |
| --- | --- | --- | --- | --- |
| Total effect of religious belief | **-.363 (.090)** | **-.380 (.092)** | **-.461 (.088)** | **-.487 (.089)** |
| Total indirect effect of religious belief | **-.149 (.035)** | **-.164 (.035)** | **-.164 (.035)** | **-.182 (.035)** |
| Indirect effect via prosocial values | *.025 (.015)* | *.028 (.016)* | .007 (.008) | .008 (.008) |
| Indirect effect via conservative identification | **-.174 (.033)** | **-.193 (.032)** | **-.170 (.034)** | **-.191 (.033)** |
| Direct effect of religious belief | **-.214 (.083)** | **-.216 (.087)** | **-.298 (.084)** | **-.305 (.087)** |
|  |  |  |  |  |
| N1/N2 | 54680 / 42 | 52413 / 40 | 54126 / 42 | 51878 / 40 |
