# Supplementary material for: “I was hungry and you gave me food”: Religiosity and attitudes toward redistribution
Source: PLoS One. 2019 Mar 22;14(3):e0214054. doi: 10.1371/journal.pone.0214054 (PMC6430507; doi:10.1371/journal.pone.0214054)
Supplement: S1 File — (DOCX) [file pone.0214054.s004.docx]

# S1 File. Equations Underlying Models 1.1-1.4 and 2.1-2.4

Our data consists of individuals (denoted as i) nested within countries (denoted as j). This is why, we used a multilevel path model in which the within-cluster and between-cluster effects are estimated separately in a single analysis (Bollen, Rabe‐Hesketh, & Skrondal, 2008, p. 452). The notation used in the following equations are largely based on (Curran & Bauer, 2007) as well as (Preacher, Rucker, & Hayes, 2007)

Models 1.1-1.4 are fixed-effects structural equation model (SEM) whereby effects of level-1 parameters remain fixed (i.e. are invariant over groups) but the intercept term β_0j_ is allowed to vary across groups.

y_ij_ = β_0j_ + β_1_x_1ij_ + β_2_x_2ij_ + β_3_m_1ij_ + β_4_m_2ij_ + β_5_m_3ij_ + r_ij_ (Models 1.1 and 1.2)

and

m_1ij_ = α_10_ + λ_11_x_1ij_ + Ɛ_1ij_

m_2ij_ = α_20_ + λ_21_x_1ij_ + Ɛ_2ij_

m_3ij_ = α_30_ + λ_31_x_2ij_ + Ɛ_3ij_ (Models 1.1 and 1.2)

where x_1_ and x_2_ refer to the independent variables, religious belief and religious social behavior that are allowed to covary with each other^[[1]](#footnote-1)^. m_1_, m_2_, and m_3_ refer to the mediators prosocial values, conservative identificaiton, and happiness, respectively. The first two mediators are predicted by religious belief (as per H1 and H2) and the third mediator is predicted by religious social behavior (as per H3). The coefficients λ_1_ and λ_2_ thus show the effect of religious belief on prosocial values and conservative identification, respectively, and λ_3_ denotes the effect of religious social behavior on happiness.

As can be seen both from the equation and from Figure 2, religious belief and religious social behavior have both direct effects and indirect effects (through the mediators) on attitudes towards redistribution. β_1_ denotes the direct effect of religious belief and β_2_ denotes the effect of religious social behavior on attitudes towards redistribution. β_3,_ β_4_, and β_5_ denote the effects of prosocial values, conservative identification, and happiness on the dependent variable, respectively. The indirect effect of each mediator could thus be calculated as a function of the effect of the independent variable on that mediator (denoted with λ) and its effect on the dependent variable (denoted with β). Thus, the indirect effect of religious belief via prosocial values would be calculated as β_3_*λ_11_, the indirect effect of religious belief via conservative identification would be calculated as β_4_*λ_21_, and the indirect effect of religious social behavior via happiness is calculated as β_5_*λ_31_.MPLUS softare calculates all direct and indirect effects along with their robust standard errors.

β_0j_ is the random intercept, which is defined as the additive function of a grand mean (ƴ_00_) and a group-level deviation from this mean (u_0j_) in Model 1.1:

β_0j_ = Ƴ_00_  + u_0j_ (Model 1.1)

While no predictors for the random intercept β_0j_ are specified in Model 1.1, Model 1.2 expands the level-2 equation to include three country-level predictors, *w*_1j_, *w*_2j_, and *w*_3j_, Social Security Laws Index, logged GDP pr capita, and religious fractionalization:

β_0j_ = ƴ_00_  + ƴ_01_*w*_1j_ + ƴ_02_*w*_2j_ + ƴ_03_*w*_3j_ + u_0j_ (Model 1.2)

Models 1.3 and 1.4 add three additional individual-level predictors to Model 1.1. These are age, gender, and education, denoted by z_1_, z_2_, and z_3_ respectively. These predictors predict both the dependent variable and the mediator variables, such that:

y_ij_ = β_0j_ + β_1_x_1ij_ + β_2_x_2ij_ + β_3_m_1ij_ + β_4_m_2ij_ + β_5_m_3ij_ + β_6_z_1ij_ + β_7_z_1ij_ + β_8_z_1ij_ + r_ij_

m_1ij_ = α_10_ + λ_11_x_1ij_ + λ_12_z_1ij_ + λ_13_z_2ij_ + λ_14_z_3ij_ + Ɛ_1ij_

m_2ij_ = α_20_ + λ_21_x_1ij_ + λ_22_z_1ij_ + λ_23_z_2ij_ + λ_24_z_3ij_ + Ɛ_2ij_

m_3ij_ = α_30_ + λ_31_x_2ij_ + λ_32_z_1ij_ + λ_33_z_2ij_ + λ_34_z_3ij_ + Ɛ_3ij_ (Models 1.3 and 1.4)

In Model 1.3, the random intercept is not predicted by any level-2 variables, while Model 1.4 adds the three country-level predictors to the specification. Therefore:

β_0j_ = Ƴ_00_  + u_0j_ (Model 1.3)

β_0j_ = ƴ_00_  + ƴ_01_*w*_1j_ + ƴ_02_*w*_2j_ + ƴ_03_*w*_3j_ + u_0j_ (Model 1.4)

Models 1.1-1.4 specify fixed coefficients for level-1 predictors to test H1, H2, and H3. Models 2.1-2.4 test H4 and H5, which state that the effect of religious belief on prosocial values and conservative identification are moderated by the level-2 variable, Social Security Laws Index (SSLI). This means that the coefficients λ_11_ and λ_21_ in Models 1.1 - 1.4 above are no longer fixed, but they vary as a function of the level-2 correlate, denoted by *w*_1_. Model 2.1 is therefore written as:

y_ij_ = β_0j_ + β_1_x_1ij_ + β_2_m_1ij_ + β_3_m_2ij_ + r_ij_

m_1ij_ = α_10_ + λ_11j_x_1ij_ + λ_12j_*w*_1j_ +Ɛ_1ij_

m_2ij_ = α_20_ + λ_21j_x_1ij_ + λ_22j_*w*_1j_ + Ɛ_2ij_

Thus prosocial values (m_1_) and conservative identification (m_2_) are now both a function of religious belief and Social Security Laws Index. Further:

β_0j_ = ƴ_00_  + ƴ_01_*w*_1j_ + u_0j_

λ_11j_ = ƴ_10_ + ƴ_11_*w*_1j_ + u_1j_

λ_21j_ = ƴ_20_ + ƴ_21_*w*_1j_ + u_2j_ (Model 2.1)

The random intercept is a function of SSLI as well as the path coefficients that predict the effect of religious belief on prosocial values and conservative identification (λ_11j_ and λ_21j_ respectively).

Model 2.2 is essentially the same as Model 2.1 but adds the two additional country-level predictors to the equation of ß_0j_ as additional country-level controls. This equation also holds for Model 2.4:

ß_0j_ = ƴ_00_  + ƴ_01_*w*_1j_ + ƴ_02_*w*_2j_ + ƴ_03_*w*_3j_ + u_0j_ (Models 2.2 and 2.4)

Models 2.3 and 2.4 adds the additional individual-level control variables age, gender, and education to the equations, such that:

y_ij_ = β_0j_ + β_1_x_1ij_ + β_2_m_1ij_ + β_4_m_2ij_ + β_6_z_1ij_ + β_7_z_1ij_ + β_8_z_1ij_ + r_ij_

m_1j_ = α_10_ + λ_11j_x_1ij_ + λ_12j_*w*_1j_ +λ_13_z_1ij_ + λ_14_z_2ij_ + λ_15_z_3ij_ +Ɛ_1ij_

m_2j_ = α_20_ + λ_21j_x_1ij_ + λ_22j_*w*_1j_ +λ_23_z_1ij_ + λ_24_z_2ij_ + λ_25_z_3ij_ +Ɛ_2ij_ (Models 2.3 and 2.4)

Equations for ß_0j_, λ_11j_, and λ_21j_ remain the same as the one for Model 2.1 for Model 2.3.

Since paths λ_11j_ and λ_21j_ denoting the effect of belief on prosocial values and conservative identification are defined as being conditional on the level-2 variable, SSLI, the MPLUS software no longer produces a specific coefficient for these paths, but rather reports the intercepts that are now predicted by SSLI. These effects are shown as SSLI 🡪 Path 1 and SSLI 🡪 Path 2 in Table 2.

The indirect effects in these models are no longer fixed, but are conditional on the values of SSLI. So although the software calculates average values for the indirect effects, it is often more instructive to inspect the indirect effects as a function of the level-2 variable, as we present in Figure 4 in the manuscript.

**References**

Bollen, K. A., Rabe‐Hesketh, S., & Skrondal, A. (2008). Structural Equation Models. In H. A. B. a. D. Collier (Ed.), *The Oxford Handbook of Political Methodology* (pp. 432-455). New York: Oxford University Press.

Curran, P. J., & Bauer, D. J. (2007). Building path diagrams for multilevel models. *Psychological Methods, 12*(3), 283-297. doi:10.1037/1082-999x.12.3.283

Kaplan, D. (2009). *Structural Equation Modeling: Foundations and Extensions* (Second ed.). California: Sage.

Kline, R. B. (2011). *Principles and Practice of Structural Equation Modeling* (Third ed.). New York: Guilford Press.

Preacher, K. J., Rucker, D. D., & Hayes, A. F. (2007). Addressing moderated mediation hypotheses: Theory, methods, and prescriptions. *Multivariate Behavioral Research, 42*(1), 185-227. doi:Doi 10.1080/00273170701341316

1. It is routine practice to specify correlations between the independent variables in structural equation models. These correlations are called unanalysed associations, because although the association is estimated by the software, they remain unanalysed in the sense that no prediction regarding why the two variables covary is put forward in the model (Kaplan, 2009; Kline, 2011, p. 16). This correlation is denoted with the curved line with two arrowheads connecting religious belief and religious social behavior in Figure 2 in the manuscript but is not presented in the equation because the effects of this correlation is not analysed in the model. [↑](#footnote-ref-1)
