# Supplementary material for: “I was hungry and you gave me food”: Religiosity and attitudes toward redistribution
Source: PLoS One. 2019 Mar 22;14(3):e0214054. doi: 10.1371/journal.pone.0214054 (PMC6430507; doi:10.1371/journal.pone.0214054)
Supplement: S2 File — (DOCX) [file pone.0214054.s005.docx]

# S2 File. Mediation and Moderation Effects across Religious Traditions

Our primary hypotheses concern the psychological mechanisms through which the belief and social behavior dimensions of religiosity affect attitudes towards redistribution. We do not produce hypotheses specific to the belonging dimension, which refers to identification with a particular organized denomination and/or a religious movement. This dimension may entail identification with a major religious tradition or with certain trends within a denomination or congregation (Smidt, Kellstedt, & Guth, 2009; Wald & Wilcox, 2006). Studying the effect of this dimension using the WVS dataset is difficult since the surveys do not collect data on identification with congregations or sects. As a result, in keeping with other cross-national studies, we operationalize this dimension as identification with major religious traditions, even though this practice may conceal variance at the level of sects, denominations, or congregations (see, for example, (Ben-Nun Bloom & Arikan, 2013).

In general, we expect the hypothesized mechanisms to mostly hold across the identifiers of major religious traditions. First, the expectation that religious beliefs are associated with prosocial values is based on evidence from cross-cultural psychology, which finds a consistent association between religious beliefs and prosocial orientations (Schwartz & Huismans, 1995). In fact, one meta-analysis that includes 21 independent samples from 15 countries and all three main monotheistic traditions found that religion is consistently positively related to benevolence across different religions, nations, and political contexts (Saroglou, Delpierre, & Dernelle, 2004). We also expect religious beliefs to be associated with conservative political identification across different religious traditions and different contexts, potentially as a result of their connection to individualism and moral judgments on hard work and effort (Stegmueller, 2013; Stegmueller, Scheepers, Rossteutscher, & de Jong, 2012) (For evidence for Christianity and Islam (Abu-Saad, 1998; Ali, 1988; Benabou & Tirole, 2006; Guiso, Sapienza, & Zingales, 2003; Pepinsky & Welborne, 2011). In addition, religious beliefs are associated with the view that big governments compete for power and prestige with religious organizations and communities (for this argument as it relates to Christianity, see (Stegmueller, 2013; Stegmueller et al., 2012; Van Kersbergen & Manow, 2009) (For the argument as it relates to Islam see (Davis & Robinson, 2012). We therefore expect the mediating effects of religious belief via conservative identification to obtain for members of the different traditions considered here.

Following the current findings in the field of positive psychology, we also expect religious social behavior to be positively linked to happiness above and beyond a specific tradition or context (Diener, Tay, & Myers, 2008; Van Cappellen, Toth-Gauthier, Saroglou, & Fredrickson, 2016). Furthermore, according to our theoretical suggestion, the psychological insurance provided by being part of a group effectively reduces threat and anxiety and is therefore crucial in raising happiness among the members of religious communities (Jost, Glaser, Kruglanski, & Sulloway, 2003).

Still, some variations due to values associated with specific teachings might emerge. For example, Catholic beliefs are argued to place more emphasis on Christian benevolence than Protestant beliefs that emphasize individual responsibility (Benabou & Tirole, 2006; Guiso et al., 2003; Kahl, 2009). Therefore, as far as Protestant identifiers are concerned, the belief dimension may not necessarily be associated with prosocial values. In addition, some scholars have argued that Muslims are motivated more by a feeling of obligation to fulfill their duty to share with others when engaging in generous actions than by feelings of compassion, which seem to be a primary motivator for Catholic identifiers (Kilinc & Warner, 2015). This may also imply that prosocial values may not be the mediating factors between religious belief and support for redistribution among Muslims. We therefore investigated to what extent the hypothesized model holds above and beyond religious belonging, by running the model separately for members of four major religious traditions – Muslims, Orthodox, Catholics, and Protestants. Since the number of observations for Jewish, Evangelical, and Hindu identifiers was too low to run multilevel path analyses, we restricted our analysis to identifiers of four religious traditions.

**Table S4a.**  **Multilevel Path Model Results for Support for Income Equality for Members of Major Religious Traditions**

|  | **MUSLIM** | **ORTHODOX** | **CATHOLIC** | **PROTESTANT** |
| --- | --- | --- | --- | --- |
| ***Religious belief mediators*** |  |  |  |  |
| Religious belief 🡪 Prosocial values | **.083 (.027)** | **.067 (.032)** | **.065 (.016)** | .017 (.024) |
| Religious belief 🡪 Conservative identification | **.236 (.076)** | **.130 (.045)** | **.188 (.036)** | **.088 (.019)** |
| ***Religious social behavior mediators*** |  |  |  |  |
| Religious social behavior 🡪 Happiness | **.038 (.020)** | **.101 (.026)** | **.059 (.017)** | .009 (.033) |
|  |  |  |  |  |
| Religious belief 🡪 DV | -.445 (.414) | -.611 (.421) | -.161 (.127) | -.050 (.110) |
| Prosocial values 🡪 DV | -.061(.435) | -.156 (.728) | **1.200 (.379)** | **1.078 (.402)** |
| Conservative identification 🡪 DV | **-.539 (.212)** | **-1.109 (.455)** | **-.832 (.204)** | **-2.134 (.334)** |
| Religious social behavior 🡪 DV | .092 (.151) | .116 (.271) | -.123 (.103) | -.243 (.116) |
| Happiness 🡪 DV | -.088 (.516) | **-1.176 (.287)** | **-.735 (.133)** | -.380 (.220) |
|  |  |  |  |  |
| Corr. (Religious belief, religious social behavior) | **.011 (.004)** | **.019 (.007)** | **.031 (.007)** | **.059 (.008)** |
|  |  |  |  |  |
| **Total, direct, and indirect effects** |  |  |  |  |
| Total effect of religious belief | **-**.577 (.426) | *-.765 (.450)* | **-.240 (.114)** | *-.219 (.127)* |
| Total indirect effect of religious belief | **-.132 (.067)** | -.154 (.100) | *-.078 (.047)* | **-.169 (.051)** |
| Indirect effect via prosocial values | -.005 (.036) | -.010 (.052) | **.078 (.029)** | .019 (.027) |
| Indirect effect via conservative identification | *-.127 (.077)* | -.144 (.095) | **-.156 (.042)** | **-.188 (.040)** |
| Direct effect of religious belief | -0.445 (.414) | -.611 (.421) | -.161 (.127) | -.050 (.110) |
| Total effect of religious social behavior | .089 (.149) | -.003 (.297) | **-.167 (.099)** | **-.246 (.120)** |
| Total indirect effect of religious social behavior | -.003 (.020) | **-.118 (.051)** | **-.044 (.017)** | -.003 (.013) |
| Indirect effect via happiness | -.003 (.020) | **-.118 (.051)** | **-.044 (.017)** | -.003 (.013) |
| Direct effect of religious social behavior | .092 (.151) | .116 (.271) | **-.123 (.103)** | **-.243 (.116)** |
| **Model fit statistics** |  |  |  |  |
| CFI / TLI / RMSEA | .977 / .942 / .005 | .859/.647 / .019 | .983 / .957 / .013 | .982 /.955 /.036 |
| Chi2 model fit for baseline model (d.f.) / p-value | 74.175 (15) / 0.000 | 133.468 (15) /  0.000 | 1035.883 (15) / 0.000 | 4307.401 (15)/ 0.000 |
| N1/N2 | 10040 / 41 | 8069 / 31 | 17529 / 45 | 9783 / 44 |

Entries are coefficients with robust standard errors in brackets. Italic entries indicate p < 0.1 (two-tailed) and bold entries indicate p < 0.05 (two-tailed).

Table S4a presents the results of the analysis for each major religious tradition along with the indirect and direct effects of religiosity dimensions on support for redistribution. The results indicate that religious belief is positively and significantly associated with prosocial values among all but Protestant identifiers. In addition, we found that the positive effect of religious belief on conservative identification is highly consistent across religious traditions. Similarly, religious social behavior is associated with higher levels of self-reported happiness among all identifiers, with the exception of Protestants. Protestant belief in hard work may explain the finding regarding the null effect of belief on prosocial orientations. Another possible explanation is that Protestants are divided into various denominations and the Protestant belief in hard work varies greatly among their members (see, for example, (Van Kersbergen & Manow, 2009) on Lutheran versus Calvinist views on social welfare) while the diversity of Protestant denominations and congregations may also explain the null effects of religious social behavior on happiness. In fact, similar null effects of religious social behavior on institutional trust for Protestants were also reported in previous studies (Ben-Nun Bloom & Arikan, 2013).

With regard to the paths from the mediator variables to the dependent variable, we found that prosocial values are positively associated with support for income equality among Catholics, Protestants, and Orthodox adherents. Conservative identification has sizable and statistically significant negative effects on the dependent variable. The calculated indirect effects conservative orientations are all statistically different from zero, with the exception of Orthodox identifiers. Finally, happiness has the expected negative effect on support for redistribution among Catholic and Orthodox identifiers.

Overall, we found that the mediating effect of conservative identification (H2) receives the most consistent support across identifiers of four major religious traditions. In addition, the hypothesis about the mediation of religious social behavior via happiness (H3) holds for Catholic and Orthodox identifiers. However, there is less empirical support for H1, which suggests that religious belief also affects support for redistribution via prosocial values. Below, we also tested whether this effect is moderated by state welfare generosity among the members of these different religious traditions as well. For this, we ran the M-SEM models separately for members of four major religious denominations in the dataset.

**Table S4b. Moderation of Paths 1 and 2 for Members of Major Religious Traditions**

|  | **MUSLIM** | **ORTHODOX*** | **CATHOLIC** | **PROTESTANT** |
| --- | --- | --- | --- | --- |
| **Within-level part of model** |  |  |  |  |
| ***Religious belief mediators*** |  |  |  |  |
| Prosocial values 🡪 DV | -.328 (.487) | -.781 (3.390) | **.945 (.342)** | **1.077 (.471)** |
| Conservative identification 🡪 DV | **-.519 (.261)** | -1.000 (2.934) | **-.832 (.212)** | **-2.074 (.316)** |
|  |  |  |  |  |
| Religious belief 🡪 DV | -.609 (.454) | -1.009 (1.678) | -.114 (.137) | -.064 (.112) |
|  |  |  |  |  |
| **Between-level part of model** |  |  |  |  |
| SSLI 🡪 Path 1 | **-.162 (.062)** | -.188 (.534) | **-.106 (.043)** | **-.123 (.028)** |
| SSLI 🡪 Path 2 | .274 (.191) | -.090 (2.019) | **.231 (.086)** | **.122 (.063)** |
|  |  |  |  |  |
| SSLI 🡪 Prosocial values | **.218 (.051)** | .229 (.464) | **.185 (.049)** | **.223 (.036)** |
| SSLI 🡪 Conservative identification | **-.386 (.144)** | -.272 (1.619) | **-.225 (.111)** | **-.163 (.052)** |
|  |  |  |  |  |
| **Total, direct, and indirect effects (Average effects)** |  |  |  |  |
| Total effect of religious belief | *-.772 (.457)* | -1.090 (2.005) | **-.236 (.116)** | -.124 (.120) |
| Total indirect effect of religious belief | -.163 (.116) | -.081 (1.299) | **-.121 (.054)** | -.060 (.049) |
| Indirect effect via prosocial values | .006 (.021) | -.012 (.171) | **.051 (.025)** | **.075 (.032)** |
| Indirect effect via conservative identification | -.170 (.111) | -.069 (1.187) | **-.173 (.046)** | **-.135 (.043)** |
| Direct effect of religious belief | -.609 (.454) | -1.009 (1.678) | -.114 (.137) | -.064 (.112) |
| **Model Fit Indices** |  |  |  |  |
| -2 x Log likelihood | 31869.49 | 15644.90 | 56682.06 | 25897.43 |
| AIC | 31913.49 | 15688.90 | 56748.06 | 25963.43 |
| BIC | 32069.14 | 15828.90 | 57000.23 | 26195.77 |
| n-adjusted BIC | 31999.23 | 15758.99 | 56895.36 | 26090.90 |
| N (Level 1 / Level 2) | 8732 / 33 | 4289/ 24 | 15394/ 35 | 8438/ 35 |

Entries are coefficients with robust standard errors in brackets. Italic entries indicate p < 0.1 (two-tailed) and bold entries indicate p < 0.05 (two-tailed).
*MLF standard errors, which tend to be highly inflated, were calculated for the models with Orthodox identifiers.

Table S4b presents the results from the M-SEM models and Figure S4 below plots the indirect effects of religious belief via prosocial values and conservative identification, and the total indirect effects of both mediators conditional on the level of state welfare generosity measured with the Social Security Laws Index (SSLI). Note that, for Orthodox identifiers, the software was unable to calculate MLR estimators so MLF estimators were calculated instead. (For possible reasons for the inability to calculate MLR estimators, see (Asparouhov & Muthén, 2010) The MLF estimators are asymptotically equivalent for correctly-specified models (Asparouhov & Muthén, 2010) and thus can be substantively interpreted. However, MLF estimators tend to overestimate standard errors, especially for models where the ratio between number of clusters and number of parameters is less than 10 (Asparouhov & Muthén, 2010). That is why we were unable to rely on statistical significance tests for this model.

For Muslim identifiers, we found that state welfare generosity has a statistically significant effect on the within-level path 1, as predicted by H4, but not on path 2 (H5). The predicted indirect effects via prosocial orientations and conservative identification are statistically not different from zero for any value of the SSLI (see also the first two panels of the top rows for both dependent variables in Figure S4). Despite finding that the SSLI significantly moderates the first path, we did not find any statistically significant conditional indirect effects for Muslims. That is, for Muslim identifiers in this dataset, the mediation of religious belief does not seem to be conditional on state welfare generosity.

For Orthodox identifiers, the coefficient for the effect of state welfare generosity on path 1 is in the expected direction (H4). As mentioned above, due to inflated standard errors, we were unable to assess the statistical significance of the moderation effect, but the substantive effect is very close to (or even higher than) the effects we observed for the moderation of path 1 for members of other religious traditions that were statistically significant (b_Muslims_= -.162; b_Catholics_= -.106; and b_Protestants_= -.123; Table S4b). However, the indirect effect of religious belief via prosocial values was not in the expected direction (see the first panel of the 2nd row in Figure S4). In addition, the effect of the SSLI on path 2 was in the unexpected direction (Table S4b), and the predicted indirect effect of religious belief via conservative identification increased as SSLI increased (see the middle panel of the 2nd row in Figure S4). Both of these results contradict H4. Thus, while we seem to have some support that the effect of religious belief via prosocial values were moderated by state welfare generosity, there is not enough evidence to establish robust findings in favor of H4 and H5 for Orthodox identifiers.

**Figure S4. Predicted Indirect Effect of Religious Belief via Prosocial Values and Conservative Identification Conditional on SSLI for Members of Major Religious Traditions**

We found that state welfare generosity has a statistically significant positive effect on path 1 and a negative and statistically significant effect on path 2. This finding provides empirical support for both H4 and H5. In addition, the indirect effect of religious belief via prosocial values and conservative identification are both in the predicted direction (see the panels in the 3rd row in Figure S4). Note that the indirect effects via conservative identification are statistically significant when the values of SSL Index are lower than .25. This suggests that the indirect effect of religious belief via conservative orientations is expected to be statistically equivalent to zero for Catholics who live in countries with low levels of state welfare generosity. We also found the total indirect effects of mediators to be statistically null unless the value of SSL Index was 0.7 or higher (see the third panel in the 3rd row of Figure A1). That is, among Catholics, the positive effect of prosocial values is expected to nullify the negative effect of conservative ideology unless state welfare generosity is very high. This finding shows that it is important to take into account the effect of context, as it indicates that religious belief has no effect on support for redistribution among Catholics in countries that do not have very generous welfare policies.

As in the case of Catholics, we found that SSLI has a statistically significant moderating effect on both path 1 and path 2 for Protestants (Table S4b). The predicted indirect effects of religious belief via prosocial values and conservative identification are also in the expected direction (see the 4th row in Figure S4). The total indirect effect of religious belief via both mediators was positive when the values of SSLI were lower than .65 (see the third panel of the 4th row in Figure S4). That is, our hypothesized model predicts religious belief to have a positive effect on support for income equality for Protestants who live in contexts with low to medium levels of state welfare generosity. After this point, the predicted indirect effects are null, as was the case with Catholics. Again, this finding attests to the importance of taking context characteristics into account to understand the relationship between religious belief and support for redistribution.

In summary, the results provide partial evidence for the moderation of religious belief paths for Muslims and the Orthodox, mostly supporting H4 but not H5, and provide full support for Protestant and Catholic identifiers.

**References**

Abu-Saad, I. (1998). Individualism and islamic work beliefs. *Journal of Cross-Cultural Psychology, 29*(2), 377-383.

Ali, A. (1988). Scaling an Islamic Work-Ethic. *Journal of Social Psychology, 128*(5), 575-583. doi:Doi 10.1080/00224545.1988.9922911

Asparouhov, T., & Muthén, B. (2010). *Multiple Imputation with Mplus*. Retrieved from <https://www.statmodel.com/download/Imputations7.pdf>

Ben-Nun Bloom, P., & Arikan, G. (2013). Religion and Support for Democracy: A Cross-National Test of the Mediating Mechanisms. *British Journal of Political Science, 43*, 375-397. doi:10.1017/S0007123412000427

Benabou, R., & Tirole, J. (2006). Belief in a just world and redistributive politics. *Quarterly Journal of Economics, 121*(2), 699-746.

Davis, N. J., & Robinson, R. V. (2012). *Claiming Society for God: Religious Movements and Social Welfare.* Bloomington, IN: Indiana University Press.

Diener, E., Tay, L., & Myers, D. G. (2008). *The Economic Vote: How Political and Economic Institutions Condition Election Results*. Cambridge: Cambridge University Press.

Guiso, L., Sapienza, P., & Zingales, L. (2003). People's opium? Religion and economic attitudes. *Journal of Monetary Economics, 50*(1), 225-282.

Jost, J. T., Glaser, J., Kruglanski, A. W., & Sulloway, F. J. (2003). Political conservatism as motivated social cognition. *Psychological Bulletin, 129*(3), 339-375. doi:10.1037/0033-2909.129.3.339

Kahl, S. (2009). Religious Doctrines and Poor Relief: A Different Causal Pathway. In K. v. K. a. P. Manow (Ed.), *Religion, Class Coalitions, and Welfare States* (pp. 267–296). Cambridge: Cambridge University Press.

Kilinc, R., & Warner, C. M. (2015). Micro-Foundations of Religion and Public Goods Provision: Belief, Belonging, and Giving in Catholicism and Islam. *Politics and Religion, 8*(4), 718-744. doi:10.1017/S1755048315000747

Pepinsky, T. B., & Welborne, B. C. (2011). Piety and Redistributive Preferences in the Muslim World. *Political Research Quarterly, 64*(3), 491-505. doi:10.1177/1065912909359404

Saroglou, V., Delpierre, V., & Dernelle, R. (2004). Values and religiosity: a meta-analysis of studies using Schwartz's model. *Personality and Individual Differences, 37*(4), 721-734.

Schwartz, S. H., & Huismans, S. (1995). Value Priorities and Religiosity in 4 Western Religions. *Social Psychology Quarterly, 58*(2), 88-107.

Smidt, C. E., Kellstedt, L. A., & Guth, J. L. (2009). The Role of Religion in American Politics: Explanatory Theories and Associated Analytical and Measurement Issues. In L. A. K. Corwin Smidt, and James L. Guth (Ed.), *Oxford Handbook on Religion and American Politics* (pp. 3-42). Oxford: Oxford University Press.

Stegmueller, D. (2013). Religion and Redistributive Voting in Western Europe. *Journal of Politics, 75*(4), 1064-1076. doi:10.1017/S0022381613001023

Stegmueller, D., Scheepers, P., Rossteutscher, S., & de Jong, E. (2012). Support for Redistribution in Western Europe: Assessing the role of religion. *European Sociological Review, 28*(4), 482-497. doi:10.1093/esr/jcr011

Van Cappellen, P., Toth-Gauthier, M., Saroglou, V., & Fredrickson, B. L. (2016). Religion and Well-Being: The Mediating Role of Positive Emotions. *Journal of Happiness Studies, 17*(2), 485-505. doi:10.1007/s10902-014-9605-5

Van Kersbergen, K., & Manow, P. (2009). *Religion, Class Coalitions, and Welfare States*. Cambridge: Cambridge University Press.

Wald, K. D., & Wilcox, C. (2006). Getting religion: Has political science rediscovered the faith factor? *American Political Science Review, 100*(4), 523-529.
