# Supplementary material for: “I was hungry and you gave me food”: Religiosity and attitudes toward redistribution
Source: PLoS One. 2019 Mar 22;14(3):e0214054. doi: 10.1371/journal.pone.0214054 (PMC6430507; doi:10.1371/journal.pone.0214054)
Supplement: S3 File — (DOCX) [file pone.0214054.s006.docx]

# S3 File. Testing of Alternative Models and Reverse Causality

Structural Equation Models (SEM) in general, and path analyses in particular, are theory-guided and confirmatory. That is, a single, theory-driven model is generally accepted or rejected based on its correspondence to the data (Kline, 2011, p. 8). Yet, there is often more than one theoretical model that can explain the relationships between variables. In such situations, the practice is to test the alternative models and compare their fit indices to those of the original model. If the fit of the alternative model is poor, there is an empirical basis for rejecting it (Kline, 2011, pp. 9, 14). Of course, testing such alternative models does not necessarily determine that the causal specifications in the proposed model are correct, but it provides an additional statistical basis for evaluating a theory-driven model (Kline, 2011). Accordingly, we ran some alternative path models and compared their fit indices to our original specification.

*Models Ia and Ib (Alternative mediators).* While recent empirical evidence shows that compassion and benevolence are associated with religious belief rather than religious social behavior (Blouin, Robinson, and Starks 2013), we wanted to test whether a model in which the social behavior dimension predicts prosocial values fits the data better. In addition, we also considered the possibility that happiness mediates the effect of religious belief due to a sense of direct connection with the divine (Byrd, Lear, & Schwenka, 2000). Accordingly, we specified two models in which we alternated the mediators of religious belief and religious social behavior. Model Ia: religious belief 🡪 prosocial values and happiness🡪 support for redistribution versus religious social behavior 🡪 conservative identification 🡪 support for redistribution; Model Ib: religious belief 🡪 happiness 🡪 support for redistribution versus religious social behavior 🡪 prosocial values and conservative identification 🡪 support for redistribution.

*Models IIa and IIb (Religiosity dimensions as mediators).* The hypothesis that religious belief predicts ideological orientations and pro-social values rather than the other way round was based on a developmental logic. In line with much of the political science literature (see Knight 2006 for a discussion), we refer to ideological orientations as “coherent and relatively stable set of beliefs or values” (Knight, 2006, p. 265); or as “belief systems and frameworks of inter-related ideas … opinions, values, and beliefs about the nature of social reality that can be grouped together under some common social theme” (Federico, 2015, p. 82). Based on this definition, ideological identification requires some comprehension of abstract concepts (Freeden, 1996). Research in developmental psychology suggests that comprehension of abstract political concepts, which are at the heart of ideological orientations, do not develop until adolescence (Piaget & Weil, 1951; Sigel & Cocking, 1977). Some even propose that it is not until the end of puberty that adolescents can grasp such concepts as society, institutions, norms, and laws (Torney-Purta, 1990). In contrast, religious beliefs are adopted early in life, and influence an individual’s everyday life from infancy onwards, given that many parents socialize children into their own religious beliefs and behaviours from a very young age (Myers, 1996). While religiosity and religious beliefs are adopted at a very young age, ideological orientations that demand more complex cognitive skills develop later in life. However, at some point in life, identification with political groups may lead an individual to become more or less devout or hold less prosocial values. In addition, one might argue that people may become more involved in religious social activities because of their ideological leanings. We therefore tested whether models in which ideology and values predict religious belief or religious social behavior better fit the data.

The first alternative model also considers the possibility that happier and more prosocial individuals tend to be more involved in religious communities (Model IIa: conservative identification 🡪 religious belief 🡪 support for redistribution versus happiness 🡪 religious social behavior 🡪 support for redistribution versus prosocial values 🡪 religious social behavior 🡪 support for redistribution); the second alternative model specifies prosocial orientations as predicting levels of religious belief (Model IIb: prosocial values 🡪 religious belief 🡪 support for redistribution versus conservative identification 🡪 religious social behavior 🡪 support for redistribution versus happiness 🡪 religious social behavior 🡪 support for redistribution).

*Models IIIa and IIIb (DV as mediator).* We also ran models in which religious belief and religious social behavior directly affect attitudes towards redistribution. The first of these models specifies support for redistribution as a mediator between religious belief and conservative identification (Model IIIa: religious belief 🡪 support for redistribution 🡪 conservative identification versus religious social behavior 🡪 support for redistribution 🡪 happiness versus religious social behavior 🡪 support for redistribution 🡪 prosocial values) while the second considers it as a mediator between religious social behavior and conservative identification (Model IIIb: religious social behavior 🡪 support for redistribution 🡪 conservative identification versus religious belief 🡪 support for redistribution 🡪 happiness and prosocial values).

*Models IVa and IVb (Further mediation checks).* Finally, since both the belief and social behavior dimensions of religiosity are closely related, both dimensions could potentially influence all three mediators. To test for this, we specified two more alternative models. Model IVa in Table A6 replaces two separate religiosity dimensions with a general religiosity index, calculated by summing the belief and behavior dimensions as an additive index. We then tested whether the effect of this simple index is mediated by prosocial values, conservative identification, and happiness at the same time. Finally, Model IVb tested whether the two separate dimensions are mediated by all three variables at the same time. That is, we specified prosocial values, conservative identification, and happiness as mediators of both religiosity dimensions at the same time.

The goodness of fit indicators of the alternative models suggest that these specifications do not fit the data as well as the hypothesized model (Table S6). In all the alternative models, CFI and TLI were lower than those of the original specifications and, in most cases, were well below the acceptable threshold of .95 or even .90 (Kline, 2011) as well as for SRMR (Hu & Bentler, 1999). These results indicate that the original specification is statistically superior, which provides further support for the hypothesized mediation effects of religiosity dimensions.

**Table S5. Fit Indices for Alternative Specifications**

|  | **Chi2 test of model fit** | **CFI** | **TLI** | **RMSEA** | **SRMR**  **(Value for within)** |
| --- | --- | --- | --- | --- | --- |
| Hypothesized model | 889.241 | .982 | .954 | .006 | .022 |
| Ia) Alternative mediators | 897.321 | .910 | .774 | .014 | .032 |
| Ib) Alternative mediators | 897.321 | .905 | .763 | .015 | .034 |
| IIa) Religiosity dimensions as mediators | 864.217 | .974 | .897 | .012 | .034 |
| IIb) Religiosity dimensions as mediators | 864.217 | .951 | .803 | .016 | .041 |
| IIIa) DV as mediator | 2084.290 | .877 | .632 | .025 | .027 |
| IIIb) DV as mediator | 2084.290 | .858 | .573 | .027 | .025 |
| IVa) Single religiosity index | 1123882.735 | .002 | -.496 | 1.186 | .253 |
| IVb) All mediators | 166.132 | .767 | -.085 | .014 | .019 |

**References**

Byrd, K. R., Lear, D., & Schwenka, S. (2000). Mysticism as a Predictor of Subjective Well-Being. *International Journal for the Psychology of Religion, 10*(4), 259–270.

Federico, C. (2015). The Structure, Foundations, and Expression of Ideology. In A. Berinsky (Ed.), *New Directions in Public Opinion* (Second ed., pp. 81-103). New York: Routledge.

Freeden, M. (1996). *Ideologies and Political Theory: A Conceptual Approach*. New York: Clarendon Press.

Hu, L. T., & Bentler, P. M. (1999). Cutoff Criteria for Fit Indexes in Covariance Structure Analysis: Conventional Criteria Versus New Alternatives. *Structural Equation Modeling-a Multidisciplinary Journal, 6*(1), 1-55. doi:10.1080/10705519909540118

Kline, R. B. (2011). *Principles and Practice of Structural Equation Modeling* (Third ed.). New York: Guilford Press.

Knight, K. (2006). Transformations of the concept of ideology in the twentieth century. *American Political Science Review, 100*(4), 619-626.

Myers, S. M. (1996). An interactive model of religiosity inheritance: The importance of family context. *American Sociological Review, 61*(5), 858-866. doi:Doi 10.2307/2096457

Piaget, J., & Weil, A. M. (1951). The Development in Children of the Idea of Homeland and Relations with Other Countries. *International Social Science Bulletin, 3*, 561-578.

Sigel, I. E., & Cocking, R. E. (1977). *Cognitive Development from Childhood to Adolescence: A Constructivist Perspective*. New York: Holt, Rinehart and Winston.

Torney-Purta, J. (1990). Youth in Relation to Social Institutes. In a. G. R. E. S.S. Feldman (Ed.), *At the Threshold: The Developing Adolescent* (pp. 457-477). Cambridge: Harvard University Press.
