# Supplementary material for: “I was hungry and you gave me food”: Religiosity and attitudes toward redistribution
Source: PLoS One. 2019 Mar 22;14(3):e0214054. doi: 10.1371/journal.pone.0214054 (PMC6430507; doi:10.1371/journal.pone.0214054)
Supplement: S4 File — (DOCX) [file pone.0214054.s007.docx]

# S4 File. Models Utilizing Different Control Variables

Parsimony is one of the crucial elements of path models (Loehlin, 1987, p. 6). Even when models control for the effects of demographic variables, it is generally recommended that not more than three of them are added (Hayduk & Littvay, 2012). It is also important that these control variables do not introduce additional measurement error (Williams, Vandenberg, & Edwards, 2009, p. 584). Accordingly, path analysis studies typically control for only a few demographic variables, such as gender and/or race that are generally measured without error. As per the common practice in path analysis, we included in the models in Tables 1 and 2 in the manuscript, gender, age, and level of education as controls. Other possible control variables, such as union membership, income, or self-ascribed socio-economic status, are generally measured with a high level of error in surveys. In particular, questions on income and socioeconomic status are subject to social desirability bias, so are often measured with error or yield high non-response rates (Tourangeau & Yan, 2007). In fact, this was the case in our data as well in that adding the income variable caused a listwise deletion of 11% of the sample (6,275 cases). Self-ascribed socio-economic status (listed as subjective social class in Table S6 below) also resulted in a loss of cases as the question was not asked in some countries.

Nevertheless, we wanted to test whether the results would still hold when using different control variables. In the summary analysis in Table S6 below, the left-hand side column lists the control variables used while the top row lists the model fit indices and the number of observations used in the analysis in Model 1.3 of Table 1 of the manuscript. These indicators are compared to the results of the alternative models using different combinations of the alternative control variables. Higher CFI and TLI values and lower RMSEA, SRMR, and chi-squared values indicate that the model better fits to the data (Kline, 2011). As can be seen from Table S7, only four of the alternative models produce model fit indices that are superior to the model used in the manuscript. Model Ia, where age, gender, and income are used as controls, produces chi-squared and SRMR values that are smaller and CFI and TLI values that are higher than the original model, showing that the alternative specification produces a better fit to the data. Similarly, models IIIa and IIId produce fit indicators that are more favorable than the original model while the fit indicators of Model IIIb are somewhat better than those of Model 1.3 presented in the manuscript. However, in all of these models, we lost cases due to listwise deletion of countries or respondents missing on the control variables. In models IIIa and IIIb, particularly, the number of countries drops to only 43 and 41, respectively, which is especially problematic given that further cases are lost in the moderation analysis because the SSLI variable is missing in some countries (Model 2). We further investigated the direct and indirect effects of religiosity indicators and found that directions and significance levels do not really change substantively (results available from the authors). Therefore, we believe that we have further support for retaining the original model specified in the manuscript.

**Table S6. Fit Indices for Models Utilizing Different Control Variables**

| **Control variables** | **Chi2 test of model fit** | **CFI** | **TLI** | **RMSEA** | **SRMR**  **(Value for within)** | **N1 / N2** |
| --- | --- | --- | --- | --- | --- | --- |
| Age, gender, education (Model 1.3 in Table 1) | 99.89 | .933 | .816 | .011 | .036 | 65278 / 49 |
| Ia) Age, gender, income | 78.604 | .946 | .850 | .010 | .030 | 58550 / 47 |
| Ib) Age, education, income | 89.187 | .930 | .806 | .011 | .036 | 58244 / 47 |
| Ic) Gender, education, income | 90.084 | .923 | .788 | .011 | .038 | 58350 / 47 |
| IIa) Age, gender, union membership | 153.619 | .901 | .706 | .014 | .033 | 62277 / 47 |
| IIb) Age, education, union membership | 153.246 | .889 | .694 | .014 | .038 | 61904 / 47 |
| IIc) Gender, education, union membership | 167.244 | .872 | .648 | .014 | .041 | 62025 / 47 |
| IId) Gender, income, union membership | 136.292 | .891 | .699 | .014 | .035 | 55642 / 45 |
| IIIa) Age, gender, subjective social class | 59.775 | .958 | .885 | .009 | .028 | 54188 / 43 |
| IIIb) Age, education, subjective social class | 74.734 | .937 | .827 | .010 | .037 | 53869 / 43 |
| IIIc) Gender, education, subjective social class | 84.384 | .922 | .786 | .011 | .039 | 54004 / 43 |
| IIId) Gender, income, subjective social class | 58.039 | .952 | .867 | .009 | .030 | 49272 / 41 |
| IVa) Education, income, subjective social class | 66.167 | .934 | .818 | .010 | .038 | 48999 / 41 |
| IVb) Education, union membership, subjective social class | 141.564 | .861 | .619 | .014 | .042 | 51860 / 42 |

**References**

Hayduk, L. A., & Littvay, L. (2012). Should Researchers Use Single Indicators, Best Indicators, or Multiple Indicators in Structural Equation Models? *BMC Medical Research Methodology, 12*. doi:<https://doi.org/10.1186/1471-2288-12-159>

Kline, R. B. (2011). *Principles and Practice of Structural Equation Modeling* (Third ed.). New York: Guilford Press.

Loehlin, J. C. (1987). *Latent variable models: An introduction to factor, pathm and structural analysis*. New Jersey: Lawrence Erlbaum Associates.

Tourangeau, R., & Yan, T. (2007). Sensitive questions in surveys. *Psychological Bulletin, 133*(5), 859-883. doi:10.1037/0033-2909.133.5.859

Williams, L. J., Vandenberg, R. J., & Edwards, J. R. (2009). Structural Equation Modeling in Management Research: A Guide for Improved Analysis. *Academy of Management Annals, 3*, 543-604. doi:10.1080/19416520903065683
