# Supplementary material for: “I was hungry and you gave me food”: Religiosity and attitudes toward redistribution
Source: PLoS One. 2019 Mar 22;14(3):e0214054. doi: 10.1371/journal.pone.0214054 (PMC6430507; doi:10.1371/journal.pone.0214054)
Supplement: S5 File — (DOCX) [file pone.0214054.s008.docx]

# S5 File. Robust Analysis of Age Effects

It is possible that the effects of age on preferences for redistribution as well as on some mediators are not linear. Since we were interested in specifying the most parsimonious models as per the requirements for path models, we only presented the linear age effects in Tables 1 and 2 in the manuscript. We have conducted a series of robust analysis where we accounted for non-linear effects by including age and age-squared in the equations (See Models S7.1 and S7.2 in Table S7 below), as well as by including age group dummies (Models S7.3 and S7.4 in Table S7). The age group dummies were based on OECD employment statistics categories: Group 1 represents respondents aged between 15 and 24, those just entering the labor market following education. Group 2 represents people aged 25 to 54 who are in their prime working lives, and Group 3 represents people aged between 55 and 64, those who are passing the peak of their career and approaching retirement. People over 65 serves as the baseline category.

We find that age-squared had a positive and statistically significant effect on prosocial values such that the positive effect of age on prosociality strengthened further with age (Models S7.1 and S7.2). Age-squared also had statistically significant effect on support for redistribution such that the negative effect of age was reversed for higher values of the variable. The addition of age-squared slightly decreased model fit: Model 1.3 in Table 1 of manuscript had CFI, TLI, and RMSEA values .933, .816, and .011 while Model S7.1 in Table S3 below has CFI, TLI, and RMSEA values of .932, .816, and .011. Similarly, Model 1.4 in Table 1 of manuscript had CFI=.946, TLI=.837, and RMSEA=.010 while Model S7.2 has CFI=.933, TLI=.835, and RMSEA=.010. More importantly, the addition of age-squared did not lead to any substantive change in the rest of the results presented in Table 1 of the manuscript.

The inclusion of age groups instead of age and age-squared slightly improved model fit in Model S7.3 such that the CFI increased from .933 (Model 1.3, Table 1 in manuscript) to .935 and RMSEA decreased from .011 (Model 1.3, Table 1 in manuscript). We found that people in all age groups were significantly less prosocial compared to the baseline of individuals over 65. Yet, age groups did not produce statistically significant effects on the other variables. Again these models also produced results that did not challenge the initial findings presented in Table 1 of the manuscript.

**Table S7. Support for Redistribution: Robust Analysis for Age Effects**

|  | **Model S7.1** | **Model S7.2** | **Model S7.3** | **Model S7.4** |
| --- | --- | --- | --- | --- |
| **Within-level part of model** |  |  |  |  |
| ***Rel. belief mediators*** |  |  |  |  |
| Religious belief 🡪 Prosocial values | **.029 (.014)** | **.031 (.015)** | **.027 (.014)** | **.029 (.015)** |
| Religious belief 🡪 Conservative identification | **.135 (.021)** | **.148 (.021)** | **.136 (.021)** | **.148 (.021)** |
| ***Religious social behavior mediators*** |  |  |  |  |
| Religious social behavior 🡪 Happiness | **.064 (.017)** | **.064 (.019)** | **.065 (.018)** | **.065 (.019)** |
|  |  |  |  |  |
| Religious belief 🡪 DV | **-.227 (.082)** | **-.239 (.091)** | **-.227 (.082)** | **-.240 (.091)** |
| Prosocial values 🡪 DV | **.630 (.291)** | **.652 (.327)** | **.614 (.288)** | *.627 (.323)* |
| Conservative identification 🡪 DV | **-1.295 (.168)** | **-1.309 (.180)** | **-1.294 (.167)** | **-1.309 (.180)** |
| Religious social behavior 🡪 DV | -.066 (.064) | -.052 (.069) | -.067 (.065) | -.054 (.070) |
| Happiness 🡪 DV | **-.551 (.117)** | **-.539 (.139)** | **-.550 (.118)** | **-.536 (.139)** |
|  |  |  |  |  |
| Corr. (Religious belief, religious social behavior | **.058 (.007)** | **.057 (.008)** | **.057 (.008)** | **.057 (.008)** |
|  |  |  |  |  |
| ***Individual-level controls*** |  |  |  |  |
| Age 🡪 Prosocial values | **.004 (.000)** | **.003 (.000)** | **-** | **-** |
| Age-squared 🡪 Prosocial values | **.000 (.000)** | **.000 (.000)** | **-** | **-** |
| Age group 1 (15-24) 🡪 Prosocial values | **-** | **-** | **-.106 (.007)** | **-.104 (.008)** |
| Age group 2 (25-54) 🡪 Prosocial values | **-** | **-** | **-.063 (.006)** | **-.061 (.006)** |
| Age group 3 (55 - 64) 🡪 Prosocial values | **-** | **-** | **-.017 (.003)** | **-.017 (.003)** |
| Gender (Male = 1) 🡪 Prosocial values | **-.024 (.003)** | **-.025 (.003)** | **-.024 (.003)** | **-.025 (.003)** |
| Low education (dummy) 🡪 Prosocial values | **-.011 (.005)** | *-.010 (.006)* | -.009 (.005) | -.009 (.006) |
|  |  |  |  |  |
| Age 🡪 Conservative identification | -.001 (.001) | -.001 (.001) | - | - |
| Age-squared 🡪 Conservative identification | .000 (.000) | .000 (.000) | **-** | **-** |
| Age group 1 (15-24) 🡪 Conservative identification | **-** | **-** | -.010 (.015) | -.009 (.016) |
| Age group 2 (25-54) 🡪 Conservative identification | **-** | **-** | -.013 (.012) | -.014 (.012) |
| Age group 3 (55 - 64) 🡪 Conservative identification | **-** | **-** | -.012 (.008) | -.008 (.008) |
| Gender (Male = 1) 🡪 Conservative identification | **.017 (.004)** | **.019 (.004)** | **.017 (.004)** | **.019 (.004)** |
| Low education (dummy) 🡪 Conservative identification | -.001 (.011) | -.004 (.012) | -.001 (.011) | -.003 (.012) |
|  |  |  |  |  |
| Age 🡪 Happiness | **-.002 (.000)** | **-.002 (.001)** | **-** | **-** |
| Age-squared 🡪 Happiness | .000 (.000) | *.000 (.000)* | - | - |
| Age group 1 (15-24) 🡪 Happiness | **-** | **-** | .041 (.021) | .038 (.022) |
| Age group 2 (25-54) 🡪 Happiness | **-** | **-** | .021 (.015) | .018 (.015) |
| Age group 3 (55 - 64) 🡪 Happiness | **-** | **-** | .010 (.008) | .004 (.008) |
| Gender (Male =1) 🡪 Happiness | -.001 (.004) | .000 (.004) | -.001 (.004) | .000 (.004) |
| Low education (dummy) 🡪 Happiness | **-.048 (.011)** | **-.050 (.011)** | **-.049 (.011)** | **-.051 (.011)** |
|  |  |  |  |  |
| Age 🡪 DV | **-.014 (.005)** | **-.016 (.006)** | - | - |
| Age-squared 🡪 DV | **.000 (.000)** | **.000 (.000)** | **-** | **-** |
| Age group 1 (15-24) 🡪 DV | **-** | **-** | .084 (.078) | .110 (.086) |
| Age group 2 (25-54) 🡪 DV | **-** | **-** | -.072 (.051) | -.064 (.055) |
| Age group 3 (55 - 64) 🡪 DV | **-** | **-** | -.023 (.045) | -.028 (.050) |
| Gender (Male =1) 🡪 DV | **-.139 (.029)** | **-.146 (.033)** | **-.141 (.029)** | **-.148 (.033)** |
| Low education (dummy) 🡪 DV | **.548 (.045)** | **.559 (.049)** | **.543 (.044)** | **.552 (.048)** |
|  |  |  |  |  |
| **Between-level part of model** |  |  |  |  |
| Social Security Laws Index | - | 1.773 (n.a.)* | - | **1.768 (.834)** |
| GDP per capita (PPP), logged | - | .194 (n.a.)* | - | .191 (.184) |
| Religious fractionalization | - | -.246 (n.a)* | - | -.259 (439) |
| **Model fit statistics** |  |  |  |  |
| CFI / TLI / RMSEA | .932/.811/.011 | .933/.835/.010 | .935/.816/.010 | .946/.837/.010 |
| Chi2 model fit for baseline model (d.f.) / p-value | 1536.753  (39) / .0000 | 1355.405  (42) / .0000 | 1725.202  (45) / .0000 | 1518.055  (48) / .0000 |
| N1/N2 | 65278 / 49 | 55028 / 40 | 65278 / 49 | 55028 / 40 |

Entries are coefficients with robust standard errors in brackets. Italic entries indicate p < 0.1 (two-tailed) and bold entries indicate p < 0.05 (two-tailed).
*The model estimation did not terminate normally due to a non-positive definite Fisher information matrix. As recommended by the software, we resolved this issue by fixing some of the model coefficients. Since our concern in this model was to test whether the effects of individual-level variables are robust to the addition of the age-squared variable, we fixed the coefficients of the level-2 control variables, based on results from Model 1.4 of Table 1 of the manuscript. This allowed the model to be estimated normally, but the standard errors of the fixed coefficients were not computed.
