# Supplementary material for: “I was hungry and you gave me food”: Religiosity and attitudes toward redistribution
Source: PLoS One. 2019 Mar 22;14(3):e0214054. doi: 10.1371/journal.pone.0214054 (PMC6430507; doi:10.1371/journal.pone.0214054)
Supplement: S6 File — (DOCX) [file pone.0214054.s009.docx]

# S8 File. Alternative Modeling Strategy to Test for Moderation Effects

We were also interested in testing the robustness of the results we found in M-SEM models. Following the suggestions and practices in the literature, we also employed a two-step strategy, which is a special case of multilevel modeling (Duch & Stevenson, 2008; Gingrich, 2014). While the multilevel approach estimates both level-1 and level-2 parameters simultaneously, the two-step approach involves analyzing each cluster separately in the first step before combining and analyzing the estimates across clusters in the second step (Gelman, 2005). This strategy can improve probabilistic inference and establish the robustness of the results, especially when there are few level-2 observations (usually <100, see (Bowers & Drake, 2005). In addition, in the two-step strategy, we can more easily control for the effects of alternative explanatory variables at the country level. This is computationally much more complex and time-consuming to do within the multilevel SEM framework so a two-step strategy is more suitable for this purpose (Bowers & Drake, 2005; Gelman, 2005).

For the two-step analysis, we first ran the path models separately for each country and saved the unstandardized coefficients and standard errors of the estimates for the path coefficients. In the second step, we used these path coefficients as dependent variables in a linear regression, which uses the information about the sampling errors in the dependent variable (Lewis & Linzer, 2005) to test whether the variation in path coefficients is explained by the key independent variable when also controlling for a number of alternative explanatory variables (Duch & Stevenson, 2008; Gingrich, 2014; Huber, Kernell, & Leoni, 2006; Jusko & Shively, 2005). Thus, these analyses also provide additional robustness checks by testing several alternative explanations of the moderation effects. Since the number of observations at the country level was not so high, we did not add too many covariates in each regression and only included the control variables with some theoretical basis.

The models in Table S8a test whether the variation in the coefficient of path 1, which shows the effect of religious belief on prosocial values, is explained by the SSLI variable (as per H4) along with a number of country-level control variables. It has been suggested that religion could be a more salient source of shared beliefs and social solidarity in countries with more difficult life conditions (Diener, Tay, & Myers, 2011; Pepinsky & Welborne, 2011). We therefore controlled for the overall level of socioeconomic development, measured by GDP per capita (logged). In addition, despite the positive associations between religion and prosocial values, there is also evidence that religious prosociality is often limited, applies mostly to proximal targets, and varies by context (Saslow et al., 2013; Shariff & Norenzayan, 2007). In fact, most prosocial behavior is observed when the religious people assume that the recipients are coreligionists (Ruffle & Sosis, 2006). We may thus expect religion to be more strongly related to prosociality in relatively more homogenous societies where most people are readily thought of as in-group members. We thus controlled for the religious fractionalization index and the Herfindahl index of religious adherence, as well as level of religious regulation by the government and religious social hostilities (Pew Government Regulation of Religion–GRI and Social Hostilities Index–SHI).

The results in Table S8a are in line with our results from the M-SEM models, which showed a positive and statistically significant effect of SLLI on the individual-level path 1. Both results provide strong empirical support for H4*,* which suggests that higher levels of government provision of social security weaken the effect of religious belief on prosocial values. All control variables that tested for alternative explanations failed to reach statistical significance.

H5 predicts that greater levels of state welfare generosity strengthen the effect of religious beliefs on conservative political identification. The models in Table S8b test H5 while also controlling for alternative explanatory variables. The rationale for H5 was that higher levels of state welfare generosity make distributive issues a more salient theme on the political agenda, thereby allowing the devout to more easily connect their values to ideological orientations. However, it is possible to propose additional explanations. First, government interference in religious freedoms could influence the effect of religious belief on conservative political identification by making religious individuals see the government as a greater threat to the power and authority of the church. Based on the findings in the literature, some religious traditions, such as Protestantism, tend to be associated with individualism and hard work values to a greater extent than some others (Stegmueller, 2013; Van Kersbergen & Manow, 2009). We therefore added religious diversity, government regulation of and social hostilities involving religion (SHI), and dummy variables for the dominant religious tradition of countries as controls in the estimated dependent variable. These models excluded Turkey, which we found to be an outlier after inspecting country-level coefficients. The coefficient for path 2 for Turkey was .518 while the next highest coefficient for the same path was only .236 for Spain (Note that the scales for variables vary between 0 and 1).

In the two-step analysis, the measure of state welfare generosity (SSLI) appears to be a robust predictor of the variation between the country-level coefficients of path 2. We did not find any evidence that religious heterogeneity, social hostilities, or government regulation moderated the effect of religious belief on conservative identification, as indicated by the large standard errors for the coefficients. However, the effect of religious belief on conservative identification was stronger in Catholic and Christian Orthodox nations than in Protestant and Muslim nations (p=.01 for Catholic nations, p=.02 for Orthodox nations, p=.23 for Protestant nations, p= .96 for Muslim nation dummies; the last model in Table S8b). This significant moderation effect may occur because Catholics are ostensibly as wary of state intervention as Calvinists (Kahl, 2009) while devout Orthodox citizens’ distrust of the state may run high in nations with a communist legacy.

Overall, the results of these analyses provide further support for the moderating effect of state welfare generosity on the mediating effect of prosocial values and conservative identification on support for redistribution, as predicted by H4 and H5.

**Table S8a. Estimated Dependent Variable Regression Results Predicting Path 1**

|  | **Model S8a.1** | **Model S8a.2** | **Model S8a.3** | **Model S8a.4** | **Model S8a.5** | **Model S8a.6** | **Model S8a.7** | **Model S8a.8** | **Model S8a.9** | **Model S8a.10** | **Model S8a.11** |
| --- | --- | --- | --- | --- | --- | --- | --- | --- | --- | --- | --- |
| SSLI | **-.093**  **(.043)** | **-** | **-.110 (.047)** | **-** | **-.107 (.051)** | **-** | **-.104 (.049)** | **-** | **-.118 (.052)** | **-** | **-.111 (.051)** |
| Logged GDP per capita, PPP | **-** | **-.015 (.007)** | .005 (.010) | **-** | .003 (.011) | **-** | .003 (.010) | **-** | .001 (.008) | **-** | .001 (.001) |
| Religious fractionalization (Alesina et al.) | **-** | **-** | **-** | .016 (.025) | .012 (.024) | - | - | **-** | - | **-** | - |
| Herfindahl index (Barro) | **-** | **-** | **-** | - | **-** | **-.050 (.028)** | -.013 (.023) | **-** | - | - | - |
| Social hostilities index (Pew) | **-** | **-** | **-** | - | **-** | - | - | .000  (.006) | -.007 (.005) | - | - |
| Government regulation index (Pew) | **-** | **-** | **-** | - | **-** | - | - | - | - | -.001 (.006) | -.007 (.006) |
| Constant | **.134 (.031)** | **.219 (.073)** | .101 (.091) | **.070 (.013)** | .114 (.088) | **.107 (.020)** | .123 (.091) | **.074 (.012)** | **.157 (.071)** | **.077 (.011)** | *.149 (.079)* |
|  |  |  |  |  |  |  |  |  |  |  |  |
| N | 34 | 38 | 33 | 39 | 33 | 40 | 33 | 40 | 33 | 40 | 33 |
| R^2^ | .23 | .16 | .21 | .00 | .24 | .07 | .24 | .00 | .31 | .00 | .28 |
| Prob > F | .038 | 0.051 | .049 | .534 | .090 | .080 | .092 | .996 | .053 | .842 | .022 |

Entries are coefficients with robust standard errors in brackets. Italic entries indicate p < 0.1 (two-tailed) and bold entries indicate p < 0.05 (two-tailed).

**Table S8b. Estimated Dependent Variable Regression Results Predicting Path 2**

|  | **Model S8b.1** | **Model S8b.2** | **Model S8b.3** | **Model S8b.4** | **Model S8b.5** | **Model S8b.6** | **Model S8b.7** | **Model S8b.8** | **Model S8b.9** |
| --- | --- | --- | --- | --- | --- | --- | --- | --- | --- |
| SSLI | **.102 (.049)** | **-** | **.102 (.047)** | **-** | **.104 (.051)** | **-** | *.098 (.050)* | - | **.095 (.044)** |
| Religious fractionalization (Alesina et al.) | **-** | -.072 (.045) | -.061  (.048) | - | - | - | - | - | - |
| Social hostilities index (Pew) | **-** | - | - | -.004 (.004) | .001 (.006) | - | - | - | - |
| Government regulation index (Pew) | **-** | **-** | **-** | **-** | **-** | -.006 (.006) | -.002 (.008) | - | - |
| Catholic nation | **-** | **-** | **-** | **-** | **-** | - | - | **.082 (.025)** | **.054 (.021)** |
| Orthodox nation | **-** | **-** | **-** | **-** | **-** | - | - | .000 (.026) | **.050 (.021)** |
| Protestant nation | **-** | **-** | **-** | **-** | *-* | **-** | **-** | .007 (.025) | .023 (.019) |
| Muslim nation | - | - | - | - | - | - | - | -.000 (.040) | -.002 (.046) |
| Constant | .021 (.032) | **.121 (.025)** | .051 (.038) | .092 (.015) | .018 (.039) | **.097 (.016)** | .027 (.038) | **.054 (.018)** | .019 (.025) |
|  |  |  |  |  |  |  |  |  |  |
| N | 34 | 39 | 34 | 40 | 34 | 40 | 34 | 40 | 34 |
| R^2^ | .11 | .06 | .15 | .01 | .11 | .02 | .12 | .33 | 0.44 |
| Prob > F | .045 | .114 | .073 | .405 | .130 | 0.26 | .147 | .008 | .008 |

Entries are coefficients with robust standard errors in brackets. Italic entries indicate p < 0.1 (two-tailed) and bold entries indicate p < 0.05 (two-tailed).

**References**

Bowers, J., & Drake, K. W. (2005). EDA for HLM: Visualization when probabilistic inference fails. *Political Analysis, 13*(4), 301-326. doi:10.1093/pan/mpi031

Diener, E., Tay, L., & Myers, D. G. (2011). The Religion Paradox: If Religion Makes People Happy, Why Are So Many Dropping Out? *Journal of Personality and Social Psychology, 101*(6), 1278-1290. doi:10.1037/a0024402

Duch, R. M., & Stevenson, R. T. (2008). *The Economic Vote: How Political and Economic Institutions Condition Election Results*. Cambridge: Cambridge University Press.

Gelman, A. (2005). Two-stage regression and multilevel modeling: A commentary. *Political Analysis, 13*(4), 459-461.

Gingrich, J. (2014). Visibility, Values, and Voters: The Informational Role of the Welfare State. *Journal of Politics, 76*(2), 565-580.

Huber, J. D., Kernell, G., & Leoni, E. L. (2006). Institutional context, cognitive resources and party attachments across democracies (vol 13, pg 365, 2006). *Political Analysis, 14*(4).

Jusko, K. L., & Shively, W. P. (2005). Applying a two-step strategy to the analysis of cross-national public opinion data. *Political Analysis, 13*(4), 327-344.

Kahl, S. (2009). Religious Doctrines and Poor Relief: A Different Causal Pathway. In K. v. K. a. P. Manow (Ed.), *Religion, Class Coalitions, and Welfare States* (pp. 267–296). Cambridge: Cambridge University Press.

Lewis, J. B., & Linzer, D. A. (2005). Estimating regression models in which the dependent variable is based on estimates. *Political Analysis, 13*(4), 345-364. doi:DOI 10.1093/pan/mpi026

Pepinsky, T. B., & Welborne, B. C. (2011). Piety and Redistributive Preferences in the Muslim World. *Political Research Quarterly, 64*(3), 491-505. doi:10.1177/1065912909359404

Ruffle, B. J., & Sosis, R. (2006). Cooperation and the in-group-out-group bias: A field test on Israeli kibbutz members and city residents. *Journal of Economic Behavior & Organization, 60*(2), 147-163. doi:10.1016/j.jebo.2004.07.007

Saslow, L. R., Willer, R., Feinberg, M., Piff, P. K., Clark, K., Keltner, D., & Saturn, S. R. (2013). My Brother's Keeper? Compassion Predicts Generosity More Among Less Religious Individuals. *Social Psychological and Personality Science, 4*(1), 31-38. doi:10.1177/1948550612444137

Shariff, A. F., & Norenzayan, A. (2007). God is watching you - Priming god concepts increases prosocial behavior in an anonymous economic game. *Psychological Science, 18*(9), 803-809.

Stegmueller, D. (2013). Religion and Redistributive Voting in Western Europe. *Journal of Politics, 75*(4), 1064-1076. doi:10.1017/S0022381613001023

Van Kersbergen, K., & Manow, P. (2009). *Religion, Class Coalitions, and Welfare States*. Cambridge: Cambridge University Press.
